# Application of CAR-T cell therapy targeting mesothelin in solid tumor treatment

**DOI:** 10.1007/s12672-024-01159-x

**Published:** 2024-07-18

**Authors:** Qiuhong Chen, Yang Sun, Hua Li

**Affiliations:** Shandong Provincial Key Laboratory of Animal Resistance Biology, College of Life Sciences, Shandong Normal University, No. 88 East Wenhua Road, Jinan, 250014 People’s Republic of China

**Keywords:** CAR-T cell therapy, Mesothelin, Solid tumors

## Abstract

Chimeric antigen receptor (CAR)-T-cell therapy is one of the most effective immunotherapies. CAR-T-cell therapy has achieved great success in the treatment of hematological malignancies. However, due to the characteristics of solid malignant tumors, such as on-target effects, off-tumor toxicity, an immunosuppressive tumor microenvironment (TME), and insufficient trafficking, CAR-T-cell therapy for solid tumors is still in the exploration stage. Mesothelin (MSLN) is a molecule expressed on the surface of various solid malignant tumor cells that is suitable as a target of tumor cells with high MSLN expression for CAR-T-cell therapy. This paper briefly described the development of CAR-T cell therapy and the structural features of MSLN, and especially summarized the strategies of structure optimization of MSLN-targeting CAR-T-cells and the enhancement methods of MSLN-targeting CAR-T cell anti-tumor efficacy by summarizing some preclinical experiment and clinical trials. When considering MSLN-targeting CAR-T-cell therapy as an example, this paper summarizes the efforts made by researchers in CAR-T-cell therapy for solid tumors and summarizes feasible treatment plans by integrating the existing research results.

## Introduction

### CAR-T

**Fig. 1:**
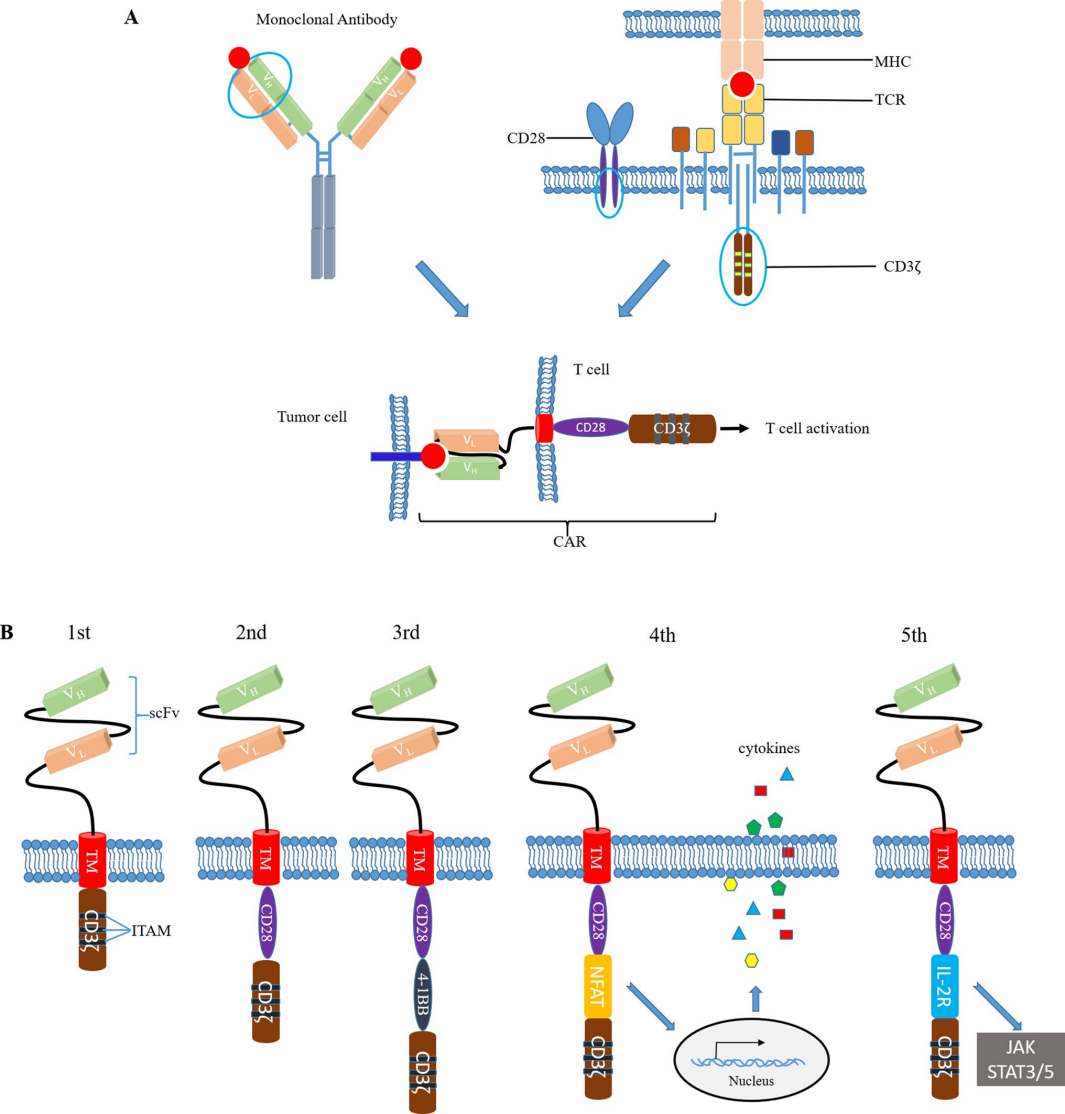
Structure and generations of CAR [5–8]. **A** The general structural components of CAR. The extracellular recognition domain was derived from the monoclonal antibody, the intracellular activation domain was derived from CD3, and the co-stimulatory domain was derived from CD28. **B** The five generations of CAR. *scFv* single chain variable fragment, *TM* transmembrane domain, *ITAM* immunoreceptor tyrosine-based activation motif. scFv combines tumor-associated antigen (TAA); transmembrane domain anchors CARs; intracellular domain delivers signals; *NFAT* nuclear factor of the activated T cell, *Jak* Janus kinase, *STAT* signal transducer and activator of transcription

Effective treatment of cancer can extend the life of cancer patients (to a certain extent) and improve their quality of life. In addition to traditional surgery and chemoradiotherapy, emerging immunotherapies have demonstrated remarkable potential in cancer treatment. CAR-T-cell therapy is a type of adoptive T-cell therapy that has rapidly developed in recent years with excellent characteristics, such as precision, speed and efficiency; moreover, it is a new tumor immunotherapy method with the potential to cure cancers [1, 2]. The structures of CARs expressed on the surface of CAR-T cells are characterized by antibodies and T-cell receptors (TCRs). After specifically recognizing target antigens on tumor cells in a major histocompatibility complex (MHC)-independent manner, CAR-T cells are activated and can effectively kill tumor cells [1, 3, 4] (Fig. 1A). The structural design of CARs is one of the key steps for the successful preparation of CAR-T cells and fundamentally determines the potency of CAR-T cells. In the past two decades, from the development of the initial concept to clinical application, the CAR structure has undergone several iterations [5, 6]. First-generation CAR contains the basic necessary structure: single-chain variable fragment (scFv), transmembrane domain (TM) and intracellular domain (CD3ζ). Because of the lack of effective intracellular activation and proliferation signals, using of T cells expressing first-generation CAR cannot achieve effective therapeutic purposes. Second-generation CAR adds a costimulatory domain (CM): CD28 or 4-1BB. Compared with the first-generation CAR-T cells, the second-generation CAR-T cells not only can specifically recognize tumor cells, but also improve proliferation and anti-tumor ability of T cells greatly in patients. It lays a solid foundation for the application of CAR-T cell therapy in cancer treatment. So far, the second-generation CAR-T has the largest proportion of applications in actual clinical treatment, and is currently a relatively mature generation. In view of the partial functional differences between CAR-T cells with CD28 (stronger killing ability) and those with 4-1BB (longer persistence), the third-generation CAR contains two co-stimulatory domains. However, with the increase of costimulatory signals, the division ability and the cell toxicity of CAR-T cells are enhanced inevitably, which increases the occurrence possibility of cytokine release syndrome (CRS) to a certain extent. Because of the character of solid tumors, some tumor cells cannot be recognized and killed by conventional CAR-T cells. Functional studies of fourth-generation CAR tend to enhance the killing efficiency of CAR-T cells on solid tumor cells, such as expressing anti-tumor cytokines in tandem, or disturbing the function of immunosuppressive factors. T cells redirected for universal cytokine killing (TRUCK T), which adds nuclear factor of the activated T cell (NFAT) to the typical CAR structure, can produce extra transgenic cytokines, such as IL-12. IL-12 can improve T cell activation and inhibit tumor angiogenesis by means of reaction with other immunomodulatory factors, and recruit other immune cells to kill cancer cells that are not recognized by CAR-T cells. Fifth-generation CAR, also known as the next generation, adds another domain to activate JAK-STAT3/5 pathway. Triple signals (CM, CD3ζ, JAK-STAT3/5 pathway) are used to activate T cell more effectively and promotes T cell proliferation and persistence [7, 8] (Fig. 1B).

At present, CAR-T-cell immunotherapy has shown strong efficacy in the treatment of hematological malignancies. In late 2017, the U.S. Food and Drug Administration (FDA) approved the first CAR-T-cell therapy for children and young adults (under 25 years of age) with relapsed or refractory acute B-cell lymphoblastic leukemia [9, 10]. Six CAR-T cell therapies have been approved by the FDA, four of which target CD19 [11–14], and two target BCMA [15, 16]. They all approve for hematologic malignancies. The success of CAR-T in the treatment of hematologic malignancies has promoted the application of CAR-T in the treatment of solid tumors. Researchers have conducted a number of preclinical experiments and clinical trials of CAR-T therapy for solid tumors. However, the therapeutic effect of CAR-T therapy is limited due to the solid tumor heterogeneity, antigen escape, CAR‑T cell trafficking and tumor infiltration and immunosuppression of TME [17]. Finding ways to overcome the barriers of solid tumor treatment is a necessary prerequisite for effectively improving the efficacy of CAR-T cell therapy for solid tumors.

### MSLN

MSLN is named for its expression in mesothelin cells [18]. In addition to normal mesothelial cells of the pleura and peritoneum, a small amount of MSLN expression can also be observed in the surface epithelium of the ovary, the tunica vaginalis, the rete testis, and the epithelial cells of the fallopian tube and tonsil [18, 19]. Moreover, MSLN is overexpressed in a variety of malignancies [19, 20], including (1) gynecologic cancers such as ovarian cancer [21–23], triple negative breast cancer (TNBC) [24–26], endometrial cancer [23, 27], and cervical cancer [23, 28]; (2) digestive system cancers such as pancreatic adenocarcinoma [21, 29–32], gastric carcinoma [21, 33, 34], and cholangiocarcinoma [5, 32, 35–37]; and (3) malignant pleural mesothelioma (MPM) [21, 25, 38–41], lung adenocarcinomas [23, 40–42] and some other squamous carcinomas of different sites of origin [5, 19] (Table 1). For tumors with high MSLN expression, it is theoretically reasonable and feasible to use MSLN as a target for CAR-T-cell immunotherapy.

**Table 1:** The positive rates of MSLN in different cancers

Cancer	Percent	Cancer	Percent
Ovarian cancer	44.4–97.3% [6, 23, 43]	PDAC	80–85% [29]
TNBC	67% [26]	Gastric carcinoma	44–78% [23, 33]
Endometrial cancer	45.5–77% [6, 43]	Cholangiocarcinoma	22% [37]
Cervical cancer	42.4% [23]	Lung adenocarcinomas	39–69% [40]
MPM	45–100% [19, 23]	Esophageal adenocarcinoma	29–46% [23]

#### Structure

The human MSLN gene is located on chromosome 16 (16P13.3), and it encodes an ~ 68 kDa precursor protein that can be cleaved into two proteins: mature MSLN, which is a membrane binding protein with a size of 40 kDa [18], and a soluble protein with a size of 31 kDa, which is known as mature megakaryocyte enhancement factor (MPF) [18, 44, 45].

**Fig. 2:**
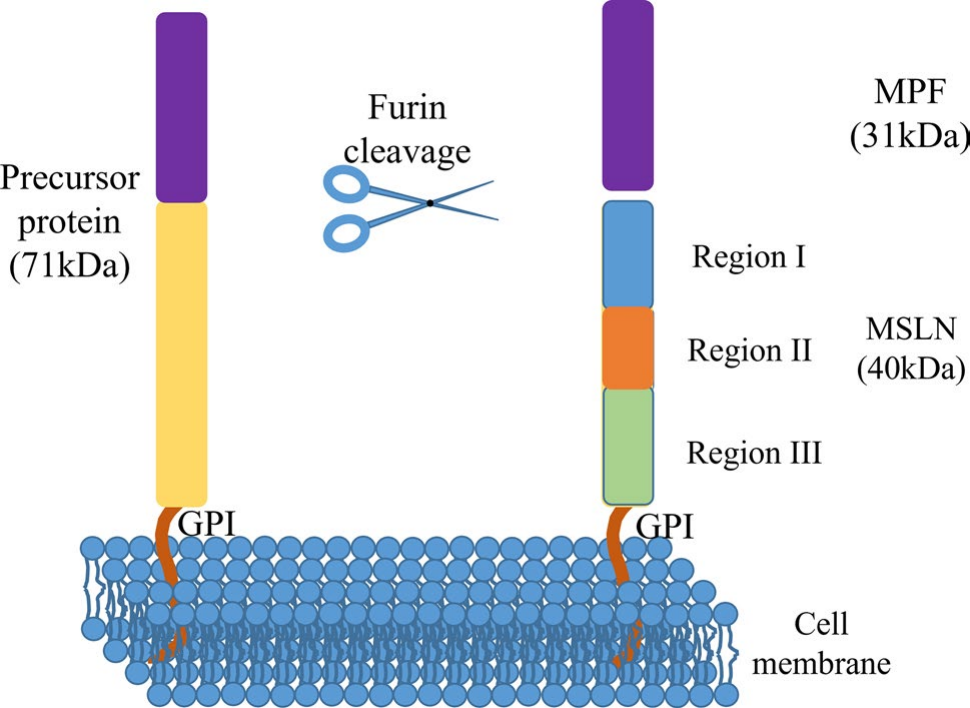
The structure of MSLN [5, 45]. The precursor protein of MSLN contains 622 amino acids and is about 68 kDa in size. It is cleaved in two proteins at arginine 295 (Arg295) by furin. The extracellular domain of MSLN consists of three contiguous regions: region I (residues 296–390), region II (391–486), and region III (487–598)

Further studies have shown that mature MSLN is a cell surface-bound glycosyl phosphatidylinositol (GPI) anchor protein with a C-terminal domain that is attached to the plasma membrane by phosphatidylinositol [5, 46]. The extracellular domain of MSLN consists of three contiguous elements: region I, region II, and region III [47] (Fig. 2). Region I is the membrane-distal region (MDR) with important binding sites, such as mucin16/carbohydrate antigen 125 (MUC16/CA125), which is associated with tumor proliferation and invasion [31, 48]. Region I is also the binding site of many immunotherapy drugs targeting MSLN for cancers [6, 49]. Region III, which is the membrane-proximal region (MPR), is the binding site of several other antibodies, such as hYP218, which can effectively prevent ineffective targeted binding caused by MSLN shedding. The elucidation of the structure of MSLN is the basis for immunotherapy-based treatment of MSLN-positive tumors [49, 50]. Zhan et al. clarified the crystal structure of MSLN; specifically, they demonstrated a compact, right-handed solenoid consisting of 24 short helices and connecting loops. These helices form a nine-layered spiral coil that resembles ARM/HEAT family proteins [51]. They also predicted the structure of three N-glycosylation sites on MSLN, which have a certain influence on the ability of T cells to recognize MSLN [51, 52].

#### Biological function

**Fig. 3:**
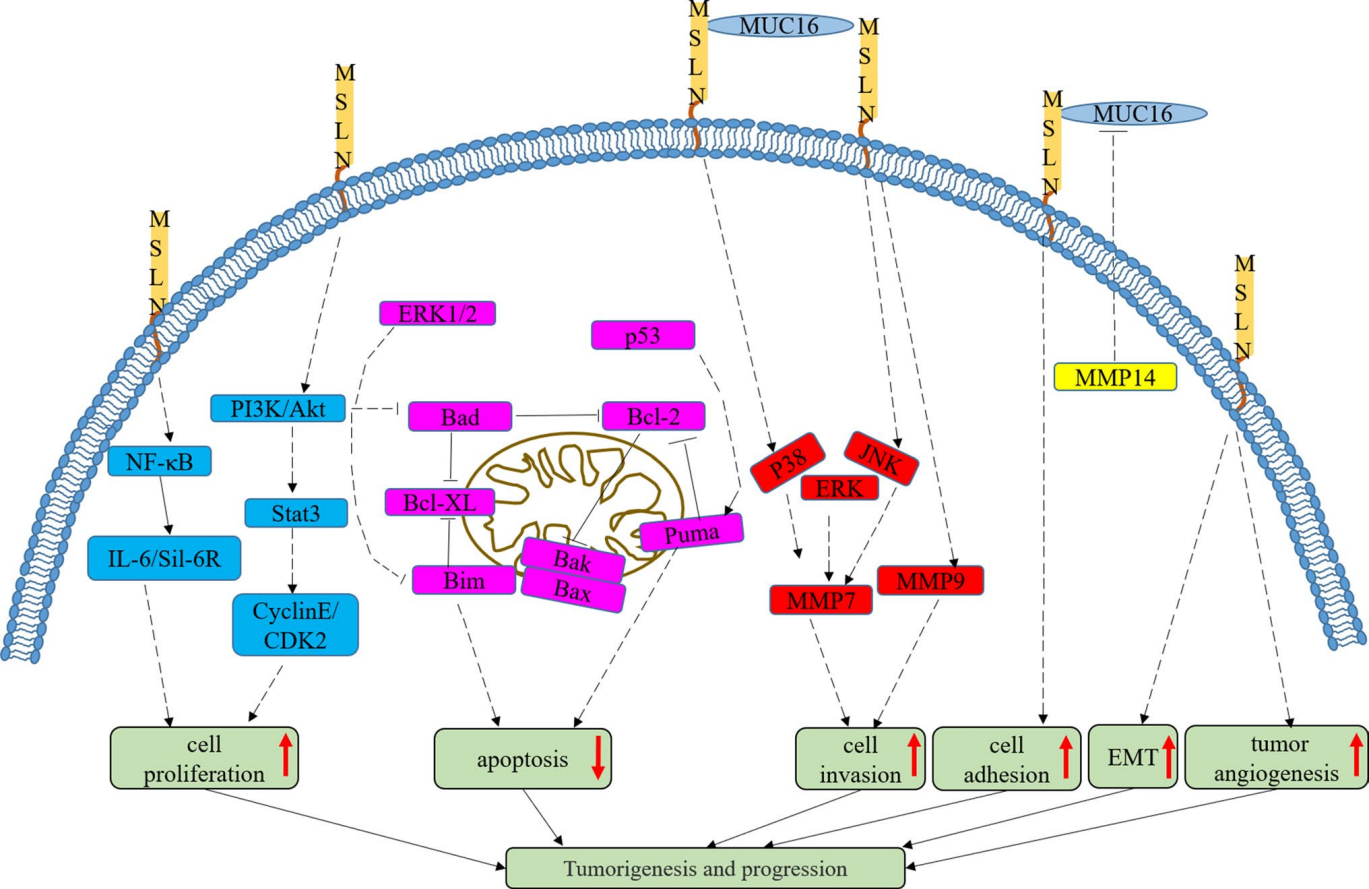
The function of MSLN in cancer cells [62, 63]. In tumor cells expressing MSLN highly, the PI3K/Akt, NF-κB, MAPK/ERK and JNK signaling pathway are activated, and the tumor EMT process and tumor microvascular formation process are also affected

The specific biological function of MSLN is unknown (except that it is not necessary for normal mouse growth) [53]. Previous studies have shown that MSLN may play a role in regulating tumor cell proliferation, apoptosis, adhesion, invasion, metastasis, and chemotherapy resistance (Fig. 3). In PC cells, MSLN overexpression can activate NF-κB and promote IL-6 expression. Cell survival and proliferation are promoted through a novel auto/paracrine interleukin-6/soluble IL-6 receptor (IL-6/sIL-6R) trans-signaling pathway [54]. The overexpression of MSLN can also activate the transcription factor Stat3 and enhance the expression of cyclin E and the cyclin E/cyclin-dependent kinase 2 complex. The transformation from the G1 to S phase is increased, thus promoting the proliferation of tumor cells and accelerating the cell cycle [55]. The overexpression of MSLN leads to sustained activation of Extracellular signal-regulated kinase 1/2 (ERK1/2), which inhibits the expression of the proapoptotic protein Bcl-2 interacting mediator of cell death (Bim). On this basis, ovarian cancer cell lines with high MSLN expression are resistant to anoikis [56]. MSLN regulates the expression of P53 up-regulated modulator of apoptosis (PUMA), BCL2-Associated X (BAX) and B-cell lymphoma-2 (BCL-2) in wt-p53 PC cells through a p53-dependent pathway, which promotes cell proliferation and inhibits apoptosis [57]. In PC cells with high MUC16 expression, the binding of MSLN and MUC16 induces the expression of matrix metalloproteinase (MMP)-7 through the p38 MAPK-dependent pathway, thus significantly enhancing the movement and invasion of PC cells [58]. MSLN promotes the expression of matrix metalloproteinase-7 (MMP-7) through the MAPK/ERK and JNK signaling pathways, thereby enhancing the migration and invasion of ovarian cancer cells [59]. MSLN promotes the expression of matrix metalloproteinase-9 (MMP-9), and the activated MMP pathway further mediates the invasion of MSLN-expressing MPM [60]. The silencing of MSLN leads to decreased expression of β-catenin, which is an important marker of epithelial–mesenchymal transition (EMT); this effect is likely to affect the invasion of tumor cells [55]. MSLN may provide a growth advantage for tumor cells in the early stage of tumor metastasis seeding [61].

When considering MSLN-targeted CAR-T-cell therapy as an example, this study summarized some of the achievements in the use of CAR-T-cell therapy for the treatment of MSLN-positive solid tumors, summarized and analyzed the common therapeutic obstacles of CAR-T-cell therapy in solid tumors, and proposed possible therapeutic strategies and feasible therapeutic programs to improve the efficacy of CAR-T-cell therapy.

## The way to improve the anti-tumor ability of anti-MSLN CAR-T cells

### Anti-MSLN CAR-T

The overexpression of MSLN in a variety of malignant tumors makes it an ideal target for immunotherapy in solid tumors. CAR-T-cell therapy targeting MSLN has been successively developed and applied. MSLN-targeting CAR-T-cell therapy has been experimentally studied in a variety of solid tumors with high MSLN expression, and the results are promising. Compared with nontransduced T cells, anti-MSLN CAR-T cells have greater antitumor cytotoxicity, cytokine secretion ability and tumor elimination ability against cancers that highly express MSLN, including TNBC [26], ovarian cancer [64], cholangiocarcinoma [65], gastric cancer [66], cervical cancer [28], bowel cancer, head and neck cancer, and colorectal cancer [67]. These in vivo and in vitro results indicate that anti-MSLN CAR-T-cell therapy is a promising treatment method for solid tumors. Currently, a large number of experiments have been performed in mouse models, and a set number of clinical trials have been performed to verify the therapeutic effect of MSLN CAR-T-cell therapy in different types of cancer with high MSLN expression. This module briefly summarizes some of the relevant studies conducted by researchers in recent years to explore the feasibility of CAR structure optimization and the effectiveness of weakening immune restriction (Table 2) to provide some references for optimizing the efficacy of anti-MSLN CAR-T-cell therapy.

**Table 2:** Studies on improving the antitumor ability of anti-MSLN CAR-T cells

Optimization	Strategy	Co-stimulatory domain	Cancer types	Year	References
Immunogenicity	Human P4 scFv	CD28	Ovarian cancer	2012	[80]
	Human P1A6E and P3F2 scFv	CD28, 4-1BB, CD28 and 4-1BB	PC	2017	[81]
	Human MS501 scFv	CD28, 4-1BB, CD28 and 4-1BB	PC	2022	[82]
MSLN binding domain	Human 15B6 scFv: binding to C-terminal of MSLN	CD28	Ovarian cancer; PC	2022	[68]
	Human hYP218: binding to region III of MSLN	4-1BB	Ovarian cancer; mesothelioma; PC	2022	[50]
	Different scFvs binding to region I, II, III or full length of MSLN	CD28 and 4-1BB	T cell lymphoma	2023	[109]
Co-stimulatory domain	Co-express the cytoplasmic domain of DAP10	CD28	Lung cancer	2019	[71]
Intracellular signal domain	CD3ζ chain containing a single ITAM (M1xx)	CD28	Ovarian cancer	2023	[73]
On-target, off-tumor	MSLN and FRα double targets; trans-signaling CAR strategy	CD28	Ovarian cancer	2013	[88]
	CEA and MSLN double targets; trans-signaling CAR strategy	4-1BB	PC	2018	[90]
	Tmod™: co-express LIR-1 inhibitory receptor targeting HLA-A*02	CD28 and 4-1BB	Cervical cancer	2022	[94]
Transfection methods	mRNA electroporation	CD28, 4-1BB, CD28 and 4-1BB	Mesothelioma	2010	[98]
	Electroporation based on piggyBac transposon system	CD28	PDAC	2018	[100]
	Electroporation based on piggyBac transposon system	CD28	Gastric cancer; ovarian cancer	2019	[52]
Method of administration	Systemic intravenous administration or local intrapleural administration	CD28	Malignant pleural carcinoma	2014	[103]
Immunosuppression	Use inhibitors of SHP-1 and DGK (T cell function inhibiting enzymes)	4-1BB	Mesothelioma	2014	[110]
	PD-1 disruption anti-MSLN CAR-T cells	4-1BB	TNBC	2019	[111]
	Knock down Tim3 using shRNA sequences	4-1BB	Ovarian cancer; cervical cancer	2021	[112]
	Co-express a small peptide called RIAD	4-1BB	PC; PDAC	2016	[113]
	Co-express A2aR shRNA sequence	4-1BB	Ovarian cancer; cervical cancer	2020	[114]
	Co-express PH20	4-1BB	Gastric carcinoma	2021	[108]
	Co-express dnTGF-βRII	4-1BB	Ovarian cancer	2023	[115]
Immune activation	Co-express IL-7 and CCL19	CD28 and 4-1BB	PDAC	2018	[116]
	Co-express IL-7 and CCL19	CD28 and 4-1BB	Malignant mesothelioma	2021	[117]
	FOLR1 and MSLN double targets and secrete IL-12	CD28 and 4-1BB	Ovarian cancer	2021	[118]
T cell migration	Co-express CCR2b	4-1BB	Mesothelioma	2011	[119]
	Co-express CCR2b or CCR4	4-1BB	NSCLC	2021	[120]

### Optimization of CAR structure

Due to the complexity of tumors (especially solid tumors), there are still considerable areas of improvement regarding the therapeutic effect of CAR-T-cell therapy. Based on this viewpoint, researchers have also performed multiangle explorations of the design and optimization of CAR structures according to the structural composition of the CAR and the structural characteristics of the MSLN.

#### scFv binding site

There are multiple molecular binding sites in region I of MSLN, including most anti-MSLN CAR-T cells. The binding of CAR-T cells may be limited in space; furthermore, the potency of CAR-T cells may be weakened or lost due to binding with shed MSLN before they reach the tumor cells or release from the cell surface along with shed MSLN. Zhang et al. reported that compared with CAR-T cells targeting region I, CAR-T cells targeting region III had the following effects: (1) expressed higher levels of lethality markers after activation; (2) produced higher levels of cytokines and had stronger tumor killing effects when cocultured with multiple MSLN-expressing cancer cells; and (3) exhibited stronger antitumor responses against gastric cancer and inhibited the growth of large ovarian tumors. CAR-T cells targeting the proximal region of MSLN have better therapeutic effects on MSLN-positive solid tumors than those targeting the distal region46. Liu et al. identified the major protease cleavage site of MSLN and produced monoclonal antibody (mAb) 15B6. mAb 15B6 strongly bound to full-length Fc-MSLN (but not to shed MSLN), targeted the C-terminal fragment of MSLN and covered all major protease cleavage sites, which inhibited MSLN shedding. The activity of 15B6 CAR-T cells was not blocked by the shed MSLN, and its antitumor activity in mice was much better than that of CAR-T cells targeting the shed MSLN [68]. Tomar et al. constructed hYP218 CAR-T cells (which bind to region III). Compared with traditional SS1 CAR-T cells targeting the MSLN I region, hYP218 CAR-T cells were more effective at killing cancer cells, and the effective target ratio to kill 50% of tumor cells (ET50) was 2–4 times lower. In ovarian cancer and mesothelioma models, one intravenous administration of hYP218 CAR-T cells improved the antitumor response and survival rate of mice, and complete tumor regression was observed. In the PC model, the expansion, functional persistence and tumor invasion of T cells were all enhanced after hYP218 CAR-T-cell administration [50]. According to the abovementioned studies, antibodies targeting the membrane proximal epitope of MSLN may have certain advantages in the treatment of MSLN-positive tumors. The binding site can indeed affect the efficacy of anti-MSLN CAR-T cells, and appropriate antibodies need to be selected according to the specific requirements of use.

#### Costimulatory domain

Moderate activation of CAR-T cells is a necessary prerequisite for their antitumor function. Intracellular CD3ζ and costimulatory domains are known to be required for CAR-T-cell activation. In addition, there are several other costimulatory molecules that function during this process. Natural killer group 2 member D (NKG2D) is a strong activating receptor for NK cells and a costimulatory receptor for T cells. NKG2D signaling is dependent on DNAX-activating protein 10 (DAP10). The phosphorylation of the—YxxM motif of DAP10 activates the downstream PI3K signaling pathway, which acts as a costimulatory signal to activate T cells [69, 70]. Zhao et al. introduced the cytoplasmic domain of DAP10 into M28z CAR-T cells by targeting MSLN. M28z10 CAR-T cells exhibited enhanced and sustained antitumor activity against MSLN+ lung cancer cells in vitro, and the secretion of Interleukin-2 (IL-2), Interferon-γ (IFN-γ), granzyme B and granulocyte–macrophage colony-stimulating factor (GM-CSF) was increased. In vivo, compared with M28z CAR-T cells, M28z10 CAR-T cells showed stronger antitumor activity in both lung cancer cell-derived xenograft (CDX) and patient-derived xenograft (PDX) mouse models. Optimization of the costimulatory domain (via the addition of DAP10) promoted the antitumor effects of traditional anti-MSLN CAR-T cells in lung cancer [71]. Thus, according to the characteristics of the co-stimulatory domain used, the addition of specific cooperating molecules can enhance the antitumor activity of CAR T cells effectively.

#### Intracellular signaling domain

The persistence of functional CAR-T cells is critical for effective killing of solid tumors. In a mouse model of B-cell malignancies, the persistence and antitumor activity of CD19 CAR-T cells were enhanced after mutation of two distal ITAMs of the CD3ζ chain [72]. Schoutrop et al. constructed MSLN-targeted CAR-T (M1xx CAR-T) cells with a CD3ζ chain containing a single ITAM (M1xx) (in which two distal ITAMs were mutated). Compared with conventional CAR-T cells, M1xx CAR-T cells can produce higher levels of IFNy, GzB and tumor necrosis factor (TNF) in vitro and exhibit a self-renewal gene signature and a low exhaustion phenotype. M1xx CAR-T cells maintained significant antitumor effects in an orthotopic ovarian cancer mouse model for a long period of time; these cells also improved the survival rate and significantly reduced the tumor burden in a peritoneal disseminated ovarian cancer mouse model [73]. The abovementioned studies suggest that TME-induced CAR-T-cell exhaustion can be partially offset by increasing the degree of CAR-T-cell activation.

In addition to the optimization of the traditional structure of MSLN-targeted CAR described above, there are also novel CAR structural designs. For example, nanobody can replace scFv [74]. Nanobodies are derived from the variable domain of heavy chain-only antibodies (HcAbs), and have the characteristics of small size, high stability, specificity and affinity. The ability and specificity of nanobodies to recognize and bind to their target antigens, as well as their solubility and stability, are similar to that of full-length mAbs [74, 75]. V_H_ and V_L_ in scFv are connected by linker, which could trigger an immune response after CAR-T cells infusion [76, 77]. However, because the nanobody does not require the presence of linker, it can effectively reduce immunogenicity. Therefore, nanobody may be a suitable component for the extracellular structure of CAR targeted MSLN.

### Toxicity weakening and technical barriers breaking

There are many difficulties in the application of CAR-T-cell therapy in the treatment of solid tumors. In addition to CAR-T-cell toxicity, there are also technical obstacles that are specific to solid tumors. These problems include (but are not limited to) the heterogeneity of target antigens, off-target effects, immunosuppression of the TME, poor trafficking and infiltration of CAR-T cells, and poor expansion and persistence of CAR-T cells [4, 5]. CAR-T cells targeting MSLN also experience many of the abovementioned difficulties. In recent years, researchers have proposed feasible improvement programs for specific problems to make CAR-T-cell therapy safer and more effective in the treatment of solid tumors.

#### Human scFv

Due to the immunogenicity of the transgene, the infusion of anti-MSLN CAR-T cells with murine scFv may trigger transgene-specific immune responses, produce human anti-mouse antibodies (HAMAs), and induce allergic reactions [78, 79]. P4, P1A6E, P3F2, MS501, and G11 are human MSLN-specific single-chain antibodies that were screened by using an antibody screening platform. P4 CAR-T cells can effectively kill MSLN-positive cancer cells, mediate bystander killing of MSLN-negative cancer cells, and mediate regression of large, established tumors in an ovarian cancer xenograft model [80]. P1A6E and P3F2 CAR-T cells were only effective against MSLN-positive PC cells. At a certain effector/target ratio, CAR-T cells with a CD28 costimulation domain had greater oncolytic ability and secreted higher levels of tumor necrosis factor alpha (TNF-α), IL-2 and IFN-γ than those with 4-1BB and could significantly inhibit the growth of tumors in a PDX mouse model of PC [81]. Similarly, MS501 CAR-T cells combined with CD28 had a more significant tumor-killing effect on PC cells. They can induce complete tumor remission, which is possibly due to the absence of off-target effects and effective infiltration [82]. G11 CAR-T cells significantly inhibited the growth of MSLN-positive ovarian tumor cells, increased the secretion of cytokines, effectively inhibited tumor growth and had relatively high antitumor activity in vivo [83]. Using of human scFv with high binding ability can avoid the production of HAMAs and kill tumor cells efficiently.

#### On-target, off-tumor toxicity

Most TAAs are also expressed in normal tissues resulting in on-target, off-tumor toxicity, which may lead to death [84]. Additionally, another phenomenon exists in single-target CAR-T-cell therapy: CAR-T cells can undergo trogocytosis during treatment, and the target antigen is transferred to the surface of T cells, thus triggering T-cell exhaustion, activity reduction and cannibalism, as well as the escape of tumor cells expressing low-density target antigen [85, 86]. Strategies involving two targets are effective methods for improving the accuracy and efficiency of the specific recognition of CAR-T cells. Lanitis et al. constructed MSLN scFv-CD3ζ and FRα scFv-CD28 CAR-T cells in trans that targeted MSLN and folate receptor-alpha (FRα), respectively, both of which are highly expressed in ovarian cancer cells [23, 87]. In dual-targeting CAR-T (dCAR-T) cells, the activation signals and costimulatory signals are physically separated, which is an approach known as the trans-signaling CAR strategy. Effective tumor killing only occurs when two CARs simultaneously recognize and bind to both target antigens. This double-specific trans-signaling CAR enhances the therapeutic effect of CAR-T cells on cancer while minimizing off-target toxicity and reducing damage to normal tissues carrying a single antigen [88]. Zhang et al. constructed dCAR-T cells targeting MSLN and carcinoembryonic antigen (CEA) [89], which were characterized by the physical separation of the signal domains of CEA-CD3ζ and MSLN-4-1BB. These dCAR-T cells were fully activated and proliferated only when cocultured with double-positive PC cells, whereas single antigen-positive tumor cells could not fully activate them. MSLN- and CEA-targeted dCAR-T cells significantly inhibited the growth of MSLN and CEA double-positive PC cells and produced a large number of cytokines with considerable persistence. However, the antitumor effect of dCAR-T cells on single-cell-positive tumor cells was not obvious [90]. Some studies have also developed methods to improve off-target toxicity for a specific group of tumor patients. Two targets can be used simultaneously, with one targeting tumor TAA and one targeting normal cell inhibitory receptors. These dual-target CAR-T cells block T-cell activity when binding to both targets at the same time and only exhibit effective cytotoxicity when recognizing tumor TAAs alone [5, 91]. Loss of heterozygosity (LOH) often occurs in cancer cells. LOH has been observed in breast cancer, colon cancer, lung cancer, brain cancer (glioblastoma) and PC [92, 93]. Tokatlian et al. constructed a dual receptor system (Tmod™) that consists of an MSLN-targeted CAR structure and a leukocyte immunoglobulin-like receptor 1 (LIR-1)-based inhibitory receptor targeting human leukocyte antigen (HLA)-A*02. This double receptor construct is designed to treat genetically defined MSLN+ cancer patients with clonal LOH of the A*02 allele. When MSLN Tmod cells recognize MSLN(+)A*02(+) normal cells, although the anti-MSLN CARs can bind to MSLN, the LIR-1 inhibitory receptor blocks the cytotoxicity of T cells to normal cells expressing the A*02 allele [94]. Tmod™, which targets CEA, also plays a positive role in the treatment of colorectal cancer [95]. CAR-T-cell therapy is expected to be applied more accurately.

After successful transfer of CARs into T cells and the generation of clonally expanded CAR-T cells, CAR-T cells are infused into patients and migrate to the tumor site to exert antitumor effects. In this process, there are many technical obstacles that affect the number and antitumor function of CAR-T cells, including (but not limited to) the following factors: (1) different CAR transduction modes vary in cost and transduction efficiency; (2) different infusion methods result in different degrees of depletion of CAR-T cells; and 3) different molecules in the tumor microenvironment have different regulatory effects on the antitumor function of CAR-T cells.

#### mRNA electroporation

To achieve stable CAR expression, viral gene transfer systems, including γ-retroviral vectors and lentiviral vectors, are generally used for CAR transduction into T cells. However, viral systems also possess several drawbacks, such as a long cycle, high cost, insertional mutagenesis and transgene silencing [96, 97]. mRNA electroporation, which is a cytoplasmic expression system, is fast and inexpensive; moreover, it can avoid some safety issues associated with viral vectors. Multiple injections of anti-MSLN mCAR-T cells were used to induce regression of large vascularized flank mesothelioma tumors in a mouse model. Intraperitoneal human-derived tumors that grew in mice for more than 50 days also exhibited significant shrinkage after multiple injections of anti-MSLN mCAR-T cells engineered from patient autologous T cells [98]. To date, many preclinical and clinical trials have demonstrated the feasibility and safety of mRNA electrotransfer. (Transposon/transposase system) The transposon/transposase system is another nonviral gene transfer system used in CAR transduction. This system has the advantages of low cost, high efficiency, simple operation and no infection risk [52, 65, 99]. He et al. produced CAR-T cells targeting MSLN (mesoCAR-T cells) by using electroporation technology based on the piggyBac transposon system. Compared with the control, mesoCAR-T cells showed rapid and powerful antitumor activity toward pancreatic ductal adenocarcinoma (PDAC) cells. After tumor remission, more mesoCAR-T cells differentiate into memory T cells with little damage to major organs [100]. Zhang et al. improved the piggyBac transposon system and constructed two anti-MSLN CAR-T cells targeting region I or region III of MSLN. Compared with mock-treated T cells, both types of CAR-T cells had a greater proportion of CD3+CD8+ T cells and memory T cells, stronger proliferation ability, increased cytokine levels, and stronger antitumor activity in vivo [52]. CAR-T cells that are constructed by using nonviral systems also have effective tumor-killing ability and persistence and can produce more memory cells. PiggyBac transposons are potential candidates for clinical conversion.

#### Infusion pattern

The infusion pattern of CAR-T cells may affect the infiltration and persistence of T cells, to some extent. Intravenously injected CAR-T cells must be circulated to the solid tumor site to function, and long-distance transport results in severe depletion of CAR-T cells. Local administration reduces the depletion and enhances the infiltration of CAR-T cells, as well as reducing systemic toxicity [101, 102]. Adusumilli et al. evaluated the effects of two routes of CAR-T-cell administration in the treatment of malignant pleural tumors. For this diffuse pleural disease, local intrapleural administration is more effective; specifically, intracranially administered CAR-T cells can effectively infiltrate into the entire thoracic tumor and have good antitumor effects, and the required dose for the eradication of pleural tumors is 30 times lower than that of intravenous injections. Although the number of CAR-T cells that accumulated in vivo was similar between these two patterns of administration, the rate of activation, expansion and differentiation of T cells induced by the antigen, as well as the antitumor efficacy and the persistence of functional T cells after intrathoracic administration, were greater than those after intravenous infusions. After intrathoracic administration, CAR-T cells can migrate out of the pleural cavity, accumulate in the external thoracic tumor via the circulation, and effectively eliminate the external thoracic tumor [103]. Local administration may have certain advantages in CAR-T-cell therapy.

#### Physical barrier

Hyaluronan (HA) and other tumor extracellular matrix (ECM) components function together to form a dense layer and prevent the infiltration of immune cells into tumor tissues [104–106]. PH20 possesses the activity of a hyaluronidase that can decompose HA into low-molecular-weight soluble HA [107]. The coexpression of sPH20-IgG2 (the secreted form of PH20) significantly enhanced the ability of anti-MSLN CAR-T cells to degrade HA in the treatment of gastric cancer with high MSLN expression; moreover, the infiltration efficiency of anti-MSLN-SP CAR-T cells in solid tumor tissues was improved. Anti-MSLN-SP CAR-T cells can shrink tumors better in a gastric cancer PDX model. sPH20-IgG2 can enhance the antitumor activity of anti-MSLN CAR-T cells against solid tumors by promoting CAR-T-cell infiltration [108]. The infiltration of CAR T cells can be effectively enhanced by reducing the hindrance of physical barrier.

#### Intrinsic T-cell inhibitory enzymes

A decrease in T-cell function is an important factor for reducing CAR-T-cell therapy efficacy. Moon et al. reported that the decrease in CAR-T-cell function was related to the increased expression of the surface inhibitory receptors programmed cell death-1 (PD1), T-cell immunoglobulin mucin 3 (TIM3), lymphocyte activation gene-3 (LAG3), and 2B4, as well as two T-cell function-inhibiting enzymes known as Src homology region 2 domain-containing phosphatase-1 (SHP-1) and diacylglycerol kinase (DGK). Inhibitors of SHP-1 and DGK can effectively enhance the killing ability of anti-MSLN CAR-T cells in vitro [110].

#### Immune checkpoint molecules

PD-1/PD-L1-mediated checkpoint inhibition limits the proliferation, activation and tumor-killing ability of CAR-T cells [121–123]. The blockage of PD-1 may reduce immunosuppression and enhance the antitumor function of CAR-T cells. Compared with those in the anti-MSLN CAR-T-cell+PD-1 mAb and anti-MSLN CAR-T control groups, PD-1-disrupted anti-MSLN CAR-T cells could better inhibit the growth of tumor cells and had a stronger ability to prevent recurrence, which may be related to the degree of T-cell exhaustion [111]. Similar to PD-1, T-cell immunoglobulin mucin 3 (Tim3) also induces CAR-T-cell exhaustion. Compared with those of anti-MSLN CAR-T cells without short hairpin RNA (shRNA) targeting segments of the human Tim3 gene, the cytotoxicity, cytokine production and proliferation of TIM3-knockdown anti-MSLN CAR-T cells were significantly enhanced in an antigen-dependent manner. The immunosuppression that is caused by high expression of Tim3 can be blocked by targeted Tim3 knockout, after which the expansion and continuous activation of tumor-infiltrating CAR-T cells can be maintained, and their antitumor ability can be restored [112]. Inhibition of the expression of immune checkpoint molecules can reduce CAR-T-cell exhaustion, and improve the anti-tumor ability of CAR-T cells.

#### Adenosine

Adenosine enrichment occurs in the hypoxic region of solid tumors, and adenosine binds to adenosine receptors on the surface of immune cells to inhibit cell proliferation and cell activity, thus interfering with the killing effect of immune system effector cells on tumor cells [124–126]. Prostaglandin E2 (PGE2) also inhibits the function of immune cells and promotes tumor growth and migration [127, 128]. Through their own G-coupled receptors, PGE2 and adenosine activate protein kinase A (PKA), which is located on immune synapses, in a cyclic adenosine monophosphate (cAMP)-dependent manner. Csk and Lck are then phosphorylated and dephosphorylated, respectively, which inhibits T-cell signaling and weakens T-cell proliferation and cytotoxicity induced by TCR [129, 130]. PKA must be anchored to a lipid raft near the cAMP-generating enzyme adenylate cyclase to function [131, 132]. Regulatory subunit I anchoring disruptor (RIAD), which is a small peptide, can shift PKA from lipid rafts and reverse the inhibition of T-cell signaling. Compared with anti-MSLN CAR-T cells, anti-MSLN CAR-T cells expressing RIAD (CAR-RIAD-T cells) showed enhanced TCR signaling, increased cytokine release, and enhanced killing of tumor cells after exposure to PGE2 or adenosine in vitro. In mouse models, CAR-RIAD-T cells can resist tumor-induced T-cell dysfunction and exhibit increased cell infiltration and antitumor efficacy. The coexpression of the RIAD peptide has certain clinical application value in the treatment of solid tumors [113]. The adenosine 2A receptor (A2aR) is a major adenosine receptor expressed on the surface of T cells [129]. Masoumi et al. constructed anti-MSLN CAR-T cells coexpressing A2aR shRNA sequences. Upon the addition of adenosine analogs, adenosine signaling, including cell proliferation, cytokine secretion and cytotoxicity, was activated, and all of the major antitumour functions of anti-MSLN CAR-T cells were inhibited. This inhibition was eliminated in CAR-T cells carrying A2aR shRNA sequences. Treatment with pharmacological antagonists of A2aR also reversed the adenosine analog-induced reduction in CAR-T-cell proliferation and cytokine response but did not have a positive effect on cell cytotoxicity [114, 133]. Liu et al. subsequently evaluated the effect of shRNA-targeted interference with A2aR on the antitumor function of anti-MSLN CAR-T cells in vitro and in vivo. When cocultured with MSLN-positive cancer cells, CAR-T cells overexpressing shRNA targeting cell-intrinsic A2aR produced a large number of cytokines and exhibited significantly increased cytotoxicity in vitro. In xenograft mouse models with high MSLN expression, A2aR-treated anti-MSLN CAR-T cells exhibited stronger antitumor activity than did anti-MSLN CAR-T cells. The abovementioned experiments further confirmed that shRNA interference with A2aR can enhance the antitumor efficacy of anti-MSLN CAR-T cells [134]. ShRNA-mediated gene expression modification may be an excellent strategy for improving CAR-T-cell function in the TME and may improve therapeutic outcomes in clinical trials.

#### Cytokines

Transforming growth factor-beta (TGF-β) plays an important role in tumor-mediated immunosuppression, and blockage of the TGF-β signaling pathway can effectively increase the cytotoxicity of T cells and enhance antitumor effects [135]. Li et al. constructed LCAR-M23 CAR-T cells that coexpressed dominant negative TGF-β receptor type II (dnTGF-βRII). The expression of dnTGF-βRII in LCAR-M23 CAR-T cells alleviated the immunosuppressive effect of TGF-β on T cells, promoted the proliferation of CAR-T cells, and improved the tumor invasion of CAR-T cells [115]. T-zone fibroblasts can produce IL-7 and CC chemokine ligand 19 (CCL19), both of which are important for the formation and maintenance of T-cell regions in lymphoid organs [136]. The coexpression of IL-7 and CCL19 has been shown to improve the infiltration and survival of CAR-T cells in mouse tumors [116]. Compared with conventional CAR-T cells, anti-MSLN CAR-T cells coexpressing IL-7 and CCL19 exhibited greater expansion and migration ability in vitro and demonstrated greater inhibition of tumor growth in PC and human mesothelioma mouse models. They could also effectively prevent the persistence and recurrence of tumors caused by a reduction in target antigen expression and maintain antitumor efficacy and target memory for a long period of time [117, 137]. IL-12 is a typical inflammatory molecule that plays a positive role in antitumor immunity [138]. CAR-T cells that simultaneously targeted folate receptor 1(FOLR1) and MSLN and secreted IL-12 were more cytotoxic to FOLR1- or MSLN-positive ovarian cells than single-target CAR-T cells and produced more cytokines, including IL-2, IFN-γ, and TNF-α. In vivo, these tandem CAR-T cells had greater tumor infiltration efficiency and stronger antitumor activity in a dose- and antigen-dependent manner after infusion [118]. These results suggest the existence of a new therapeutic strategy. The blockage of the function of immunosuppressive cytokines or the coexpression of cytokines with positive regulators of immune responses may reverse tumor-derived immunosuppression in the local TME and enhance the cytotoxicity of CAR-T cells to improve the antitumor functions of CAR-T cells.

#### T cell migration

The effective trafficking of circulating CAR-T cells is an important prerequisite for their antitumor activity. Chemokines play an important role in the trafficking of immune cells to tumor tissues [139]. CCL2 is highly expressed in a variety of malignant tumor cells, but its receptors (CCR2b and CCR4) are only slightly expressed on the surface of activated CAR-T cells that target tumor antigens [119]. The coexpression of CCR2b and CCR4, which are the receptors of CCL2, can improve the homing ability and antitumor effect of CAR-T cells in the treatment of hematologic malignancies [140, 141]. Similar results have been reported for the treatment of solid tumors. Compared with conventional anti-MSLN CAR-T cells, CAR-T cells coexpressing CCR2b exhibited increased CCL2-induced calcium flux and transport, increased levels of IL-2, IFN-γ, and TNF-α, and enhanced cytotoxicity against MSLN+ tumor cells in vitro. In vivo, they exhibited stronger migration and infiltration ability and increased antitumor function in malignant mesothelioma and NSCLC models [119, 120]. The coexpression of functional chemokine receptors may improve the efficiency of CAR-T-cell trafficking.

## Combination therapy

Current animal experiments and preclinical experiments have shown that single CAR-T-cell therapy has limited therapeutic effects on solid tumors. The combination of other therapies can increase the effectiveness of solid tumor treatment and has good application prospects.

### CD40 agonist

CD40 is a member of the TNF receptor superfamily. Activation of CD40 can effectively improve the antitumor ability of T cells [142]. Agonists targeting CD40 that are used alone or in combination with other methods have achieved positive results in cancer treatment [143, 144]. Zhang et al. used the piggyBac transposon to generate CAR-T cells targeting MSLN region III while secreting anti-CD40 antibodies (Meso3-CD40 CAR-T cells). After target antigen stimulation, Meso3-CD40 CAR-T cells secreted more cytokines, exhibited greater cytotoxicity and a greater proportion of central memory T cells and exhibited a stronger antitumor response than meso3 CAR-T cells [145]. CD40 agonists are also suitable for combination with CAR-T-cell therapy.

### PD-1 blocking therapy

Immunosuppression mediated by immunosuppressive molecules such as PD-1 promotes the exhaustion of CAR-T cells and weakens the antitumor function of CAR-T cells. PD-1 blocking antibodies can restore anti-MSLN CAR-T-cell function [122]. The use of genetic engineering methods to block the expression of PD-1 in CAR-T cells can achieve the same effect [111, 112]. PD-1 blockade therapy can not only effectively eliminate the inhibition of CAR-T-cell function but also improve the overall tumor killing ability of the immune system. At present, PD-1 blocking antibodies are widely used in clinical trials of MSLN-targeted CAR-T-cell therapy [146–149].

### Oncolytic adenoviruses

Oncolytic adenoviruses (OAds) can replicate and proliferate continuously in cancer cells and kill cancer cells but have little effect on normal cells [150]. When anti-MSLN CAR-T cells combined with OAd-TNFa-IL2 (an oncolytic adenovirus expressing TNF-α and IL-2) were applied to human PDA xenotransplantation mice, the activity, proliferation, tumor invasion and killing ability of the resulting anti-MSLN CAR-T cells were enhanced. Significant tumor regression was also observed in the syngeneic immunocompetent mouse PDA model. Ad-mTNFa-mIL2 can secrete chemokines such as MCP-1, CXCL-10 and RANTES, which recruit CAR-T cells and host T cells into tumors, alter host tumor immune status via M1 polarization of macrophages, and increase the maturation of dendritic cells. These findings suggest that cytokine-armed oncolytic adenoviruses can effectively enhance the efficacy of CAR-T-cell therapy while activating innate antitumor activity [151]. The TGF-β-targeting oncolytic adenovirus rAd.sT is an OAd that expresses soluble TGF-β receptor II-Fc. rAd.sT can effectively reduce TGF-β signaling and inhibit tumor growth and metastasis. Moreover, rAd.sT can directly lyse TNBC cells and has obvious antitumor effects in the initial stage. The combined application of rAd.sT and anti-MSLN CAR-T cells increased the production of IL-6, IL-12 and other cytokines and resulted in a stronger antitumor response in a TNBC model [152]. Oncolytic viruses may be an option for combination therapy.

### Chemotherapy

Lymphodepletion before CAR-T-cell treatment can reduce endogenous lymphocytes, remove immunosuppressive cells (such as regulatory T cells), improve the TME, and increase access to cytokines that facilitate T-cell expansion, which can promote the expansion and engraftment of CAR-T cells and significantly improve the antitumor response of CAR-T cells [153, 154]. The combined use of oxaliplatin (Ox) and cyclophosphamide (Cy) can cause immunogenic cell death (ICD) in cancer cells, induce T-cell infiltration, control tumor growth, and trigger systemic immune responses in patients [151, 155]. Using an NSCLC genetically engineered mouse model that simulated the initiation, progression and treatment response of human lung cancer, Srivastava et al. reported that the TME changed after Ox/Cy treatment; in particular, the expression of CCR5 and CXCR3 was activated, which enhanced CAR-T-cell infiltration and increased tumor sensitivity to checkpoint blockade [156]. The camptothecin-derived drug irinotecan is often used in combination with other drugs to treat cancer patients [157]. CAR_R47, which targets the MSLN I region, has good antitumor activity. The combined use of CAR_R47 and irinotecan significantly enhanced the infiltration of CD4+ and CD8+ T cells and effectively eliminated tumor cells in the H9 CDX model. The tumors in the colorectal cancer (CRC) PDX model were completely resolved. The combination of CAR_R47 with the chemotherapy drug irinotecan can significantly improve the antitumor effect of CAR_R47, which can be considered a therapeutic strategy for the treatment of colorectal cancer [109].

## Anti-MSLN CAR-T-cell clinical trial

Many clinical trials of anti-MSLN CAR-T-cell therapy for MSLN-positive solid tumors have been conducted to explore the safety and efficacy of CAR-T-cell therapy. More than 50 anti-MSLN CAR-T-cell-related clinical trials have been registered on the ClinicalTrials.gov website (https://clinicaltrials.gov/). To date, only a few of these clinical trials have been completed, and most are in recruitment or in progress. Several articles with published clinical data were selected for an overview in this review (Table 3).

**Table 3:** Clinic trials of anti-MSLN CAR-T cell therapy for solid tumors

NCT number	Treatment strategy	Cancer type	Year	Phase	Status	References
NCT01355965	mRNA electroporation anti-MSLN CAR-T cells containing murine-derived scFv	PDAC; MPM	May 1, 2011	Phase I	Completed	[79, 159]
NCT02159716	Anti-MSLN CAR-T cells containing murine-derived scFv + cyclophosphamide pretreatment	PDAC; MPM; Ovarian Cancer	Jun 1, 2014	Phase I	Completed	[158]
NCT01897415	Autologous anti-MSLN mRNA CAR-T cells	PDAC	Jul 1, 2013	Phase I	Completed	[160]
NCT03198546	Anti-MSLN CAR-T cells co-expressing IL-7 and CCL19	Advanced PC	July 1, 2017	Phase I	Recruiting	[137]
NCT02414269	Switch regional delivery of anti-MSLN CAR-T cells with suicide switch + pembrolizumab + cyclophosphamide	MPM	May 1, 2015	Phase I/II	Active, not recruiting	[148]
NCT03545815	Anti-MSLN CAR-T cells knocking out PD-1	Solid Tumor	Mar 19, 2018	Phase I	Unknown	[149]
NCT03615313	Anti-MSLN CAR-T cells secreting PD-1 antibodies + apatinib	Advanced metastatic ovarian cancer	2018/8/6	Phase I/II	Unknown	[147, 162]

### Immunogenicity

To evaluate the safety of anti-MSLN CAR-T-cell therapy containing murine-derived scFv, Maus et al. transfected MSLN mRNA into T cells via electroporation and evaluated the targeted nontumor toxicity of meso-RNA-CAR-T cells. Four patients (1 PC, 3 MPM) received autologous T-cell therapy. Patients in this clinical study were given repeated transfusions of meso-RNA-CAR-T cells to ensure efficacy. One subject experienced an allergic reaction and cardiac arrest within minutes after the third infusion, and this toxicity could not be controlled by terminating the T-cell infusion. This is the first description of a clinical allergic reaction caused by CAR-modified T cells, and it was most likely due to CAR-specific IgE antibodies. The results suggested that the potential immunogenicity of murine-derived CARs may be a safety concern for mRNA CARs, especially when intermittent dosing is used (NCT01355965) [79]. Haas et al. came to a similar conclusion; specifically, they observed that anti-MSLN CAR-T cells with mouse scFv were well tolerated and expanded in the blood of all of the patients but had limited clinical activity, which was possibly due to immunogenicity, as human anti-chimeric antibodies (HACA) were detected in the blood of 8/14 patients (NCT02159716) [158].

### mRNA electroporation

Beatty et al. established a clinical platform to construct anti-MSLN CAR-T cells by using mRNA electroporation, which may avoid off-target effects. A phase I clinical trial showed that anti-MSLN mRNA CAR-T-cell immunotherapy was feasible and safe. No significant adverse effects, such as pleurisy, pericarditis, or peritonitis, occurred in two of the presented case reports (1 PC, 1 MPM). Additionally, mRNA-engineered T cells have a limited lifespan in patients, thus allowing for potential off-target toxicity to be assessed in a controlled manner. In patients with advanced cancer, short-lived CAR-T cells can also induce epitope diffusion and mediate antitumor activity. These findings supported the development of tumor therapy strategies based on mRNA-electroporated CAR-T cells (NCT01355965) [159]. Beatty et al. also evaluated the safety and efficacy of autologous MSLN mRNA CAR-T cells in six patients with chemotherapy refractory metastatic PDAC. Patients who received continuous intravenous infusion of anti-MSLN CAR-T cells did not develop CRS, neurological symptoms during treatment, or dose-limiting toxicity. Two patients were in stable condition by the end of the study, and their progression-free survival (PFS) times were 3.8 months and 5.4 months. The metabolic activity volume remained stable in 3 patients, and MSLN expression decreased by 69.2% in 1 patient. These results suggested that anti-MSLN mRNA CAR-T cells have potential antitumor activity, and their safety, feasibility, and therapeutic potential for the treatment of PDAC patients have been validated (NCT01897415) [160].

### Cyclophosphamide pretreatment

Haas et al. investigated the safety and activity of MLSN CAR-T cells that were generated by using lentiviral transduction in patients with ovarian cancer, MPM, and PDAC. Moreover, they also investigated the effect of cyclophosphamide preconditioning on the efficacy of CAR-T-cell therapy. A total of 15 patients with chemotherapy-resistant cancers participated in this clinical trial. Fifteen patients were treated with a single infusion of CAR-T-meso cells (with or without cyclophosphamide). The results showed that the CAR-T-cell combination was well tolerated by the CAR-T cells. One patient with PDAC who developed dose-limiting toxicity (grade 4, septicemia) received a 1–3 × 10^7^/m^2^ CAR-T-cell infusion without cyclophosphamide. The best overall response was stable disease (11/15 patients). CAR-T cells expanded in the blood after infusion and peaked on Days 6–14; however, the duration was short. Two months after infusion, this phenomenon was already undetectable in more than half of the patients. However, it was still detectable in two patients after 6 months of infusion. Cyclophosphamide pretreatment enhanced CAR-T-cell amplification, and this improvement lasted for 28 days (NCT02159716) [158].

### IL-7 and CCL19 co-expression

Pang et al. used anti-MSLN CAR-T cells coexpressing human IL-7 and CCL19 (7 × 19 CAR-T cells) to initiate a phase I clinical trial in patients with advanced PC who were highly expressing MSLN. CAR-T cells (7 × 19) were intravenously administered every 1–2 months. After five common intravenous infusions over 240 days, the tumors of the patients were almost completely eliminated. No grade 2–4 adverse events or major complications occurred. The incorporation of IL-7 and CCL19 into CAR-T cells significantly enhanced their antitumor activity against human solid tumors (NCT03198546) [137].

### PD-1 blocking therapy

Fang et al. constructed aPD1-MSLN-CAR-T cells that can secrete PD-1 antibodies. The safety and efficacy of aPD1-MSLN-CAR-T cells were investigated in 10 patients with relapsed/refractory solid cancer who had failed standard therapy. Patients were treated with aPD1-MSLN-CAR-T cells for ≥ 2 cycles. After treatment, patients generally experienced adverse reactions, such as mild fatigue and fever, as well as grade 1–2 CRS, and there were no neurologic symptoms. Treatment responses varied among the 10 patients. Specifically, two patients achieved a partial response (PR), 4 patients developed stable disease (SD), and 4 patients experienced disease progression (PD). The Kaplan‒Meier method estimated a median PFS of 97 days (95% CI [13, 180]). Thus, modified anti-MSLN CAR-T cells expressing PD-1 antibodies are safe for treating solid tumors (NCT03615313) [147]. Adusumilli et al. conducted the first phase I study of locally delivered autologous MSLN-targeted CAR-T-cell therapy for the treatment of MPM. After determining the safe dose of CAR-T cells for intrapleural injection and verifying the ability of PD-1 blockade to enhance the effect of CAR-T-cell therapy in mice, 18 MPM patients were given pembrolizumab after cyclophosphamide treatment and anti-MSLN CAR-T-cell infusion. This combination was observed to be safe and effective. The median overall survival with CAR-T-cell infusion was 23.9 months (1-year overall survival, 83%). Eight patients were stable for ≥ 6 months. A PET scan demonstrated a complete metabolic response in 2 patients. The expression of PD-L1 was upregulated after CAR-T-cell infusion, and the use of an anti-PD-L1 antibody may effectively restore the function of CAR-T cells and endogenous T cells recruited to the tumor site (NCT02414269) [139]. Wang et al. further explored the impact of PD-1 blockade in CAR-T-cell therapy by knocking out PD-1. By combining CRISPR-Cas9 with lentiviral transduction technology, MPTK-CAR-T cells, which are a type of MSLN-targeted 28ζ CAR-T-cell (P4 scFv was selected) with PD-1, were generated. A total of 15 MSLN-positive patients with refractory or relapsing tumors received one or more intravenous transfusions of MPTK-CAR-T cells at a low starting dose (1–2 × 10^5^/kg) in a dose-progressive manner. CRS, neurotoxicity or autoimmune reactions were not observed in the trial; however, there was off-target toxicity. MPTK CAR-T cells can metastasize effectively into tumors, but this effect did not last longer than 1 month. Among the 15 patients, two patients had tumors that shrunk by nearly 20%, seven patients achieved SD at 3–4 weeks after infusion, and the median PFS of these seven patients was 7.1 weeks. The median overall survival (OS) was 3.0 months for 15 patients and 4.9 months for the previously mentioned 7 patients. These results preliminarily confirm the feasibility and safety of CRISPR-Cas9-mediated destruction of PD-1 CAR-T cells for the treatment of solid tumors (NCT03545815) [149]. PD-1/PD-L1 blockade can effectively improve the ability of CAR-T cells to recognize and kill tumor cells.

### Apatinib

Apatinib is a class of molecular drug that can inhibit the proliferation and formation of tumor blood vessels, with a relatively clear effect being observed on some metastatic and advanced cancers; additionally, it has also been used in combination with some immunotherapies [161]. One patient with advanced metastatic ovarian cancer achieved only short-term remission after treatment with apatinib. Fang et al. generated αPD-1-MesoCAR-T cells, which encode the scFv of MSLN and a full-length antibody against PD-1, after which they combined CAR-T-cell therapy with apatinib to treat this patient. After αPD-1-MesoCAR-T-cell infusion, both the expression of PD-1 antibody and the number of CAR-T cells in the peripheral blood of patients were significantly increased; moreover, the number of metastatic nodules was reduced, and the patient's condition achieved remission, with only a grade 1 adverse reaction being observed. The application of anti-MSLN CAR-T cells secreting PD-1 antibodies combined with apatinib can improve treatment outcomes and prolong PFS in patients with chemotherapy refractory ovarian cancer. This effect may be attributed to the synergistic effect of CAR-T cells, PD-1 antibodies, and angiogenesis inhibitor drugs (NCT03615313) [162].

Currently, the clinical trials of CAR-T-cell therapy that have been registered have different research priorities. In addition to the abovementioned published studies, other studies have focused on the following ideas: (1) testing the therapeutic effect of different types of anti-MSLN CAR-T cells and determining the maximum tolerated dose, including UCLM802 (NCT05775666 and NCT05848999), LD013 (NCT05372692), A2B694 (NCT06051695), LCAR-M23 (NCT04562298), and MCY-M11 (NCT03608618), among other studies; (2) combination therapy, including chemotherapy drugs (NCT03608618 and NCT02792114), PD-1 blocking therapy (NCT05373147 and NCT05089266), and oncolytic virus (NCT05057715), among other studies; (3) comparison of infusion methods, including systemic infusion and local injection (NCT04577326 and NCT03608618), vascular interventional therapy or intratumoral injection (NCT02959151 and NCT02706782), endoscopic ultrasound intervention (NCT06054308), among other studies; (4) safety and effectiveness of different transfection methods, including electroporation (NCT04981691) and lentiviral transduction (NCT03054298), among other studies; and (5) partial remission of TME inhibition, including dnTGFβRII (NCT05166070 and NCT05141253).

## Conclusion

This study systematically reviewed the structure and function of MSLN and summarized the exploration and partial results of CAR-T cells targeting MSLN in the treatment of solid tumors in three aspects: the feasibility of applying anti-MSLN CAR-T cells to different types of solid tumors, the possibility of CAR structure optimization, and the effectiveness of toxicity impairment and technical barriers for breakthrough. CAR-T-cell immunotherapy is undoubtedly one of the most effective methods to treat cancer and has good application potential in the treatment of hematological malignancies. However, due to the characteristics of solid tumors, such as the heterogeneity of tumor antigens, the inhibitory effect of the TME, and poor T-cell trafficking, the efficacy of anti-MSLN CAR-T cells in the treatment of MSLN-positive solid tumors is not satisfactory. To improve the therapeutic effect, overcome the shortcomings of CAR-T-cell therapy in the treatment of solid tumors, and realize the rapid application of CAR-T-cell therapy in the clinical treatment of solid tumors, a large number of researchers have been striving to conduct more in-depth research for decades. The treatment designs and engineering strategies that are used in ongoing clinical trials, including target selection, off-target toxicity reduction, immune activation promotion, immunosuppression reduction, and TME improvement, also differ among different patients. Although the therapeutic effects of anti-MSLN CAR-T cells are different in different types and stages of solid tumors, they are generally effective. In addition, combination therapy has shown advantages over single CAR-T-cell therapy in vivo and in clinical trials. The combination of chemotherapy drugs, immunosuppressants and other treatment methods can effectively improve the treatment efficacy of anti-MSLN CAR-T cells. Anti-MSLN CAR-T-cell therapy combined with other therapies is expected to become the main treatment for MSLN-positive malignant solid tumors in the future.

## Data Availability

Not applicable.

## Abbreviations

A2aR: Adenosine 2A receptor
BAX: BCL2-associated X
BCL-2: B-cell lymphoma-2
Bim: Bcl-2 interacting mediator of cell death
cAMP: Cyclic adenosine monophosphate
CAR-T: Chimeric antigen receptor
CCL19: CC chemokine ligand 19
CCR2b: CC chemokine receptor 2b
CCR4: CC chemokine receptor 4
CD: Cluster of differentiation
CDX: Cell derived xenograft
CEA: Carcinoembryonic antigen
CRC: Colorectal cancer
CRS: Cytokine release syndrome
Csk: C-terminal Src kinase
Cy: Cyclophosphamide
DAP10: DNAX-activating protein 10
DGK: Diacylglycerol kinase
dnTGF-βRII: Dominant negative TGF-β receptor type II
ECM: Extracellular matrix
EMT: Epithelial–mesenchymal transition
ERK: Extracellular signal-regulated kinase 1/2 (ERK1/2)
FOLR1: Folate receptor 1
FRα: Folate receptor-alpha
GM-CSF: Granulocyte-macrophage colony-stimulating factor
GPI: Glycosyl phosphatidylinositol
HA: Hyaluronan
HACA: Human anti-chimeric antibodies
HAMA: Human anti-mouse antibodies
HLA: Human leukocyte antigen
ICD: Immunogenic cell death
IFN-γ: Interferon-γ
IgG: Immunoglobulin G
IL-2: Interleukin-2
IL-6/sIL-6R: Interleukin-6/soluble IL-6 receptor
LAG3: Lymphocyte activation gene-3
Lck: Lymphocyte-specific protein-tyrosine kinase
LIR-1: Leukocyte immunoglobulin-like receptor 1
LOH: Loss of heterozygosity
mAb: Monoclonal antibody
MAPK/JNK: Mitogen-activated protein kinase/c-Jun NH2-terminal kinase
MDR: Membrane-distal region
MHC: Major histocompatibility complex
MMP-7: Matrix metalloproteinase-7
MMP-9: Matrix metalloproteinase-9
MPF: Mature megakaryocyte enhancement factor
MPM: Malignant pleural mesothelioma
MPR: Membrane-proximal region
MSLN: Mesothelin
MUC16/CA125: Mucin16/carbohydrate antigen 125
NKG2D: Natural killer group 2 member D
NF-κB: Nuclear factor kappa-B
OAds: Oncolytic adenoviruses
OAd-TNFa-IL2: Oncolytic adenovirus expressing TNF-α and IL-2
Ox: Oxaliplatin
OS: Overall survival
PD: Disease progression
PD1/PD-L1: Programmed cell death-1/programmed cell death-ligand 1
PDAC: Pancreatic ductal adenocarcinoma
PDX: Patient derived xenograft
PFS: Progression-free survival
PGE2: Prostaglandin E2
PKA: Protein kinase A
PR: Partial response
PUMA: P53 up-regulated modulator of apoptosis
RIAD: Regulatory subunit I anchoring disruptor
scFv: Single-chain variable fragment
SD: Stable disease
SHP-1: Src homology region 2 domain-containing phosphatase-1
shRNA: Short hairpin RNA
TAA: Tumor associated antigen
TCR: T cell receptor
TGF-β: Transforming growth factor
Tim3: T-cell immunoglobulin mucin 3
TME: Tumor microenvironment
TNBC: Triple negative breast cancer
TNF: Tumor necrosis factor
TNF-α: Tumor necrosis factor alpha

## References

[1] Batlevi CL, Matsuki E, Brentjens RJ, Younes A (2016). Novel immunotherapies in lymphoid malignancies. Nat Rev Clin Oncol.

[2] Tokarew N, Ogonek J, Endres S, Bergwelt-Baildon MV, Kobold S (2019). Teaching an old dog new tricks: next-generation CAR T cells. Brit J Cancer.

[3] Gattinoni L, Powell DJ, Rosenberg SA, Restifo NP (2006). Adoptive immunotherapy for cancer: building on success. Nat Rev Immunol.

[4] Sterner RC, Sterner RM (2021). CAR-T cell therapy: current limitations and potential strategies. Blood Cancer J.

[5] Zhai X, Mao L, Wu M, Liu J, Yu S (2023). Challenges of anti-mesothelin CAR-T-cell therapy. Cancers.

[6] Zhang G, Li T, Han S (2021). Mesothelin-targeted CAR-T cells for adoptive cell therapy of solid tumors. Arch Med Sci.

[7] Smirnov S, Mateikovich P, Samochernykh K, Shlyakhto E (2024). Recent advances on CAR-T signaling pave the way for prolonged persistence and new modalities in clinic. Front Immunol.

[8] Dejenie TA, G/Medhin MT, Terefe GD, Admasu FT, Tesega WW, Abebe EC (2022). Current updates on generations, approvals, and clinical trials of CAR T-cell therapy. Hum Vaccines Immunother..

[9] Labanieh L, Majzner RG, Mackall CL (2018). Programming CAR-T cells to kill cancer. Nat Biomed Eng.

[10] Lancet T (2017). CAR T-cells: an exciting frontier in cancer therapy. Lancet.

[11] FDA. Package insert-KYMRIAH. 2022. https://www.fda.gov/media/107296/download. Accessed 13 Dec 2022.

[12] FDA. Package insert-YESCARTA. 2022. https://www.fda.gov/media/108377/download. Accessed 13 Dec 2022.

[13] FDA. Package insert-TECARTUS. 2022. https://www.fda.gov/media/140409/download. Accessed 13 Dec 2022.

[14] FDA. Package insert-BREYANZI. 2022. https://www.fda.gov/media/145711/download. Accessed 13 Dec 2022.

[15] FDA. Package insert-ABECMA. 2021. https://www.fda.gov/media/147055/download. Accessed 13 Dec 2022.

[16] FDA. Package insert-CARVYKTI. 2022. https://www.fda.gov/media/156560/download. Accessed 13 Dec 2022.

[17] Chen X, Li P, Tian B, Kang X (2022). Serious adverse events and coping strategies of CAR-T cells in the treatment of malignant tumors. Front Immunol.

[18] Chang K, Pastan I (1996). Molecular cloning of mesothelin, a differentiation antigen present on mesothelium, mesotheliomas, and ovarian cancers. Proc Natl Acad Sci USA.

[19] Ordonez NG (2003). Application of mesothelin immunostaining in tumor diagnosis. Am J Surg Pathol.

[20] Hassan R, Thomas A, Alewine C, Le DT, Jaffee EM, Pastan I (2016). Mesothelin immunotherapy for cancer: ready for prime time?. J Clin Oncol.

[21] Inaguma S, Wang Z, Lasota J, Onda M, Czapiewski P, Langfort R (2017). Comprehensive immunohistochemical study of mesothelin (MSLN) using different monoclonal antibodies 5B2 and MN-1 in 1562 tumors with evaluation of its prognostic value in malignant pleural mesothelioma. Oncotarget.

[22] Magalhaes I, Fernebro J, Abd Own S, Glaessgen D, Corvigno S, Remberger M (2020). Mesothelin expression in patients with high-grade serous ovarian cancer does not predict clinical outcome but correlates with CD11c^+^ expression in tumor. Adv Ther.

[23] Weidemann S, Gagelmann P, Gorbokon N, Lennartz M, Menz A, Luebke AM (2021). Mesothelin expression in human tumors: a tissue microarray study on 12,679 tumors. Biomedicines.

[24] Parinyanitikul N, Blumenschein GR, Wu Y, Lei X, Chavez-Macgregor M, Smart M (2013). Mesothelin expression and survival outcomes in triple receptor negative breast cancer. Clin Breast Cancer.

[25] Suzuki T, Yamagishi Y, Einama T, Koiwai T, Yamasaki T, Fukumura-Koga M (2020). Membrane mesothelin expression positivity is associated with poor clinical outcome of luminal-type breast cancer. Oncol Lett.

[26] Tchou J, Wang L, Selven B, Zhang H, Conejo-Garcia J, Borghaei H (2012). Mesothelin, a novel immunotherapy target for triple negative breast cancer. Breast Cancer Res Tr.

[27] Dainty LA, Risinger JI, Morrison C, Chandramouli GV, Bidus MA, Zahn C (2007). Overexpression of folate binding protein and mesothelin are associated with uterine serous carcinoma. Gynecol Oncol.

[28] He Y, Li X, Yin C, Wu Y (2020). Killing cervical cancer cells by specific chimeric antigen receptor-modified T cells. J Reprod Immunol.

[29] Hassan R, Laszik ZG, Lerner M, Raffeld M, Postier R, Brackett D (2005). Mesothelin is overexpressed in pancreaticobiliary adenocarcinomas but not in normal pancreas and chronic pancreatitis. Am J Clin Pathol.

[30] Nahm CB, Turchini J, Jamieson N, Moon E, Sioson L, Itchins M (2019). Biomarker panel predicts survival after resection in pancreatic ductal adenocarcinoma: a multi-institutional cohort study. Eur J Surg Oncol.

[31] Shimizu A, Hirono S, Tani M, Kawai M, Okada K, Miyazawa M (2012). Coexpression of MUC16 and mesothelin is related to the invasion process in pancreatic ductal adenocarcinoma. Cancer Sci.

[32] Swierczynski SL, Maitra A, Abraham SC, Iacobuzio-Donahue CA, Ashfaq R, Cameron JL (2004). Analysis of novel tumor markers in pancreatic and biliary carcinomas using tissue microarrays. Hum Pathol.

[33] Einama T, Homma S, Kamachi H, Kawamata F, Takahashi K, Takahashi N (2012). Luminal membrane expression of mesothelin is a prominent poor prognostic factor for gastric cancer. Brit J Cancer.

[34] Shin SJ, Park S, Kim MH, Nam CM, Kim H, Choi YY (2019). Mesothelin expression is a predictive factor for peritoneal recurrence in curatively resected stage III gastric cancer. Oncologist.

[35] Nomura R, Fujii H, Abe M, Sugo H, Ishizaki Y, Kawasaki S (2013). Mesothelin expression is a prognostic factor in cholangiocellular carcinoma. Int Surg.

[36] Yu L, Feng M, Kim H, Phung Y, Kleiner DE, Gores GJ (2010). Mesothelin as a potential therapeutic target in human cholangiocarcinoma. J Cancer.

[37] Ordóñez NG (2003). Value of mesothelin immunostaining in the diagnosis of mesothelioma. Modern Pathol.

[38] Chang K, Pai L, Pass H, Pogrebniak H, Tsao M, Pastan I (1992). Monoclonal antibody K1 reacts with epithelial mesothelioma but not with lung adenocarcinoma. Am J Surg Pathol.

[39] Scales SJ, Gupta N, Pacheco G, Firestein R, French DM, Koeppen H (2014). An antimesothelin-monomethyl auristatin E conjugate with potent antitumor activity in ovarian, pancreatic, and mesothelioma models. Mol Cancer Ther.

[40] Kachala SS, Bograd AJ, Villena-Vargas J, Suzuki K, Servais EL, Kadota K (2014). Mesothelin overexpression is a marker of tumor aggressiveness and is associated with reduced recurrence-free and overall survival in early-stage lung adenocarcinoma. Clin Cancer Res.

[41] Kushitani K, Takeshima Y, Amatya VJ, Furonaka O, Sakatani A, Inai K (2007). Immunohistochemical marker panels for distinguishing between epithelioid mesothelioma and lung adenocarcinoma. Pathol Int.

[42] Thomas A, Chen Y, Steinberg SM, Luo J, Pack S, Raffeld M (2015). High mesothelin expression in advanced lung adenocarcinoma is associated with KRAS mutations and a poor prognosis. Oncotarget.

[43] O'Hara M, Stashwick C, Haas AR, Tanyi JL (2016). Mesothelin as a target for chimeric antigen receptor-modified T cells as anticancer therapy. Immunotherapy.

[44] Urwin D, Lake RA (2000). Structure of the mesothelin/MPF gene and characterization of its promoter. Mol Cell Biol Res Commun.

[45] Klampatsa A, Dimou V, Albelda SM (2021). Mesothelin-targeted CAR-T cell therapy for solid tumors. Expert Opin Biol Ther.

[46] Pastan I, Hassan R (2014). Discovery of mesothelin and exploiting it as a target for immunotherapy. Cancer Res.

[47] Kaneko O, Gong L, Zhang J, Hansen JK, Hassan R, Lee B (2009). A binding domain on mesothelin for CA125/MUC16. J Biol Chem.

[48] Hilliard T (2018). The impact of mesothelin in the ovarian cancer tumor microenvironment. Cancers.

[49] Zhang Y, Phung Y, Gao W, Kawa S, Hassan R, Pastan I (2015). New high affinity monoclonal antibodies recognize non-overlapping epitopes on mesothelin for monitoring and treating mesothelioma. Sci Rep.

[50] Tomar S, Zhang J, Khanal M, Hong J, Venugopalan A, Jiang Q (2022). Development of highly effective anti-mesothelin hYP218 chimeric antigen receptor T Cells with increased tumor infiltration and persistence for treating solid tumors. Mol Cancer Ther.

[51] Zhan J, Lin D, Watson N, Esser L, Tang WK, Zhang A (2023). Structures of cancer antigen mesothelin and its complexes with therapeutic antibodies. Cancer Res Commun.

[52] Zhang Z, Jiang D, Yang H, He Z, Liu X, Qin W (2019). Modified CAR T cells targeting membrane-proximal epitope of mesothelin enhances the antitumor function against large solid tumor. Cell Death Dis.

[53] Bera TK, Pastan I (2000). Mesothelin is not required for normal mouse development or reproduction. Mol Cell Biol.

[54] Bharadwaj U, Marin-Muller C, Li M, Chen C, Yao Q (2011). Mesothelin confers pancreatic cancer cell resistance to TNF-a-induced apoptosis through Akt/PI3K/NF-κB activation and IL-6/Mcl-1 overexpression. Mol Cancer.

[55] Bharadwaj U, Li M, Chen C, Yao Q (2008). Mesothelin-induced pancreatic cancer cell proliferation involves alteration of cyclin E via activation of signal transducer and activator of transcription protein 3. Mol Cancer Res.

[56] Uehara N, Matsuoka Y, Tsubura A (2008). Mesothelin promotes anchorage-independent growth and prevents anoikis via extracellular signal-regulated kinase signaling pathway in human breast cancer cells. Mol Cancer Res.

[57] Zheng C, Jia W, Tang Y, Zhao H, Jiang Y, Sun S (2012). Mesothelin regulates growth and apoptosis in pancreatic cancer cells through p53-dependent and -independent signal pathway. J Exp Clin Canc Res.

[58] Chen S, Hung W, Wang P, Paul C, Konstantopoulos K (2013). Mesothelin binding to CA125/MUC16 promotes pancreatic cancer cell motility and invasion via MMP-7 activation. Sci Rep.

[59] Chang MC, Chen CA, Chen PJ, Chiang YC, Chen YL, Mao TL (2012). Mesothelin enhances invasion of ovarian cancer by inducing MMP-7 through MAPK/ERK and JNK pathways. Biochem J.

[60] Servais EL, Colovos C, Rodriguez L, Bograd AJ, Nitadori J, Sima C (2012). Mesothelin overexpression promotes mesothelioma cell invasion and MMP-9 secretion in an orthotopic mouse model and in epithelioid pleural mesothelioma patients. Clin Cancer Res.

[61] Avula LR, Rudloff M, El-Behaedi S, Arons D, Albalawy R, Chen X (2020). Mesothelin enhances tumor vascularity in newly forming pancreatic peritoneal metastases. Mol Cancer Res.

[62] Faust JR, Hamill D, Kolb EA, Gopalakrishnapillai A, Barwe SP (2022). Mesothelin: an immunotherapeutic target beyond solid tumors. Cancers.

[63] Montemagno C, Cassim S, Pouyssegur J, Broisat A, Pagès G (2020). From malignant progression to therapeutic targeting: current insights of mesothelin in pancreatic ductal adenocarcinoma. Int J Mol Sci.

[64] Carpenito C, Milone MC, Hassan R, Simonet JC, Lakhal M, Suhoski MM (2009). Control of large, established tumor xenografts with genetically retargeted human T cells containing CD28 and CD137 domains. Proc Natl Acad Sci USA.

[65] Katayama H, Yasuchika K, Miyauchi Y, Kojima H, Yamaoka R, Kawai T (2017). Generation of non-viral, transgene-free hepatocyte like cells with piggyBac transposon. Sci Rep.

[66] Lv J, Zhao R, Wu D, Zheng D, Wu Z, Shi J (2019). Mesothelin is a target of chimeric antigen receptor T cells for treating gastric cancer. J Hematol Oncol.

[67] Zhang Q, Liu G, Liu J, Yang M, Fu J, Liu G (2021). The antitumor capacity of mesothelin-CAR-T cells in targeting solid tumors in mice. Mol Ther Oncolytics.

[68] Liu X, Onda M, Watson N, Hassan R, Ho M, Bera TK (2022). Highly active CAR T cells that bind to a juxtamembrane region of mesothelin and are not blocked by shed mesothelin. Proc Natl Acad Sci USA.

[69] Bauer S, Groh V, Wu J, Steinle A, Phillips JH, Lanier LL (1999). Activation of NK cells and T cells by NKG2D, a receptor for stress-inducible MICA. Science.

[70] Prajapati K, Perez C, Rojas LB, Burke B, Guevara-Patino JA (2018). Functions of NKG2D in CD8^+^ T cells: an opportunity for immunotherapy. Cell Mol Immunol.

[71] Zhao R, Cheng L, Jiang Z, Wei X, Li B, Wu Q (2019). DNAX-activating protein 10 co-stimulation enhances the anti-tumor efficacy of chimeric antigen receptor T cells. Oncoimmunology.

[72] Feucht J, Sun J, Eyquem J, Ho Y, Zhao Z, Leibold J (2019). Calibration of CAR activation potential directs alternative T cell fates and therapeutic potency. Nat Med.

[73] Schoutrop E, Poiret T, El-Serafi I, Zhao Y, He R, Moter A (2023). Tuned activation of MSLN-CAR T cells induces superior antitumor responses in ovarian cancer models. J Immunother Cancer.

[74] Kozani PS, Naseri A, Mirarefn SMJ, Salem F, Nikbakht M, Bakhshi SE (2022). Nanobody-based CAR-T cells for cancer immunotherapy. Biomark Res.

[75] Muyldermans S (2013). Nanobodies: natural single-domain antibodies. Annu Rev Biochem.

[76] Shah NN, Fry TJ (2019). Mechanisms of resistance to CAR T cell therapy. Nat Rev Clin Oncol.

[77] Jin BK, Odongo S, Radwanska M, Magez S (2023). Nanobodies: a review of generation, diagnostics and therapeutics. Int J Mol Sci.

[78] Lamers HJ, Sleijfer S, Vulto AG, Kruit WHJ, Kliffen M, Debets R (2006). Treatment of metastatic renal cell carcinoma with autologous T-lymphocytes genetically retargeted against carbonic anhydrase IX: first clinical experience. J Clin Oncol.

[79] Maus MV, Haas AR, Beatty GL, Albelda SM, Levine BL, Liu X (2013). T cells expressing chimeric antigen receptors can cause anaphylaxis in humans. Cancer Immunol Res.

[80] Lanitis E, Poussin M, Hagemann IS, Coukos G, Sandaltzopoulos R, Scholler N (2012). Redirected antitumor activity of primary human lymphocytes transduced with a fully human anti-mesothelin chimeric receptor. Mol Ther.

[81] Jiang H, Song B, Wang P, Shi B, Li Q, Fan M (2017). Efficient growth suppression in pancreatic cancer PDX model by fully human anti-mesothelin CAR-T cells. Protein Cell.

[82] Lee HH, Kim I, Kim UK, Choi SS, Kim TY, Lee D (2022). Therapeutic efficacy of T cells expressing chimeric antigen receptor derived from a mesothelin-specific scFv in orthotopic human pancreatic cancer animal models. Neoplasia.

[83] Chen J, Hu J, Gu L, Ji F, Zhang F, Zhang M (2023). Anti-mesothelin CAR-T immunotherapy in patients with ovarian cancer. Cancer Immunol Immunother.

[84] Morgan RA, Yang JC, Kitano M, Dudley ME, Laurencot CM, Rosenberg SA (2010). Case report of a serious adverse event following the administration of T cells transduced with a chimeric antigen receptor recognizing ERBB2. Mol Ther.

[85] Hamieh M, Dobrin A, Cabriolu A, van der Stegen SJC, Giavridis T, Mansilla-Soto J (2019). CAR T cell trogocytosis and cooperative killing regulate tumour antigen escape. Nature.

[86] Schoutrop E, Renken S, Micallef Nilsson I, Hahn P, Poiret T, Kiessling R (2022). Trogocytosis and fratricide killing impede MSLN-directed CAR T cell functionality. Oncoimmunology.

[87] Kalli KR, Oberg AL, Keeney GL, Christianson TJH, Low PS, Knutson KL (2008). Folate receptor alpha as a tumor target in epithelial ovarian cancer. Gynecol Oncol.

[88] Lanitis E, Poussin M, Klattenhoff AW, Song D, Sandaltzopoulos R, June CH (2013). Chimeric antigen receptor T cells with dissociated signaling domains exhibit focused antitumor activity with reduced potential for toxicity in vivo. Cancer Immunol Res.

[89] Imaoka H, Mizuno N, Hara K, Hijioka S, Tajika M, Tanaka T (2016). Prognostic impact of carcinoembryonic antigen (CEA) on patients with metastatic pancreatic cancer: a retrospective cohort study. Pancreatology.

[90] Zhang E, Yang P, Gu J, Wu H, Chi X, Liu C (2018). Recombination of a dual-CAR-modified T lymphocyte to accurately eliminate pancreatic malignancy. J Hematol Oncol.

[91] Fedorov VD, Themeli M, Sadelain M (2013). PD-1- and CTLA-4-based inhibitory chimeric antigen receptors (iCARs) divert off-target immunotherapy responses. Sci Transl Med.

[92] Beroukhim R, Mermel CH, Porter D, Wei G, Raychaudhuri S, Donovan J (2010). The landscape of somatic copy-number alteration across human cancers. Nature.

[93] Hwang MS, Mog BJ, Douglass J, Pearlman AH, Hsiue EH, Paul S (2021). Targeting loss of heterozygosity for cancer-specific immunotherapy. Proc Natl Acad Sci USA.

[94] Tokatlian T, Asuelime GE, Mock J, Diandreth B, Sharma S, Toledo Warshaviak D (2022). Mesothelin-specific CAR-T cell therapy that incorporates an HLA-gated safety mechanism selectively kills tumor cells. J Immunother Cancer.

[95] Sandberg ML, Wang X, Martin AD, Nampe DP, Gabrelow GB, Li CZ (2023). A carcinoembryonic antigen-specific cell therapy selectively targets tumor cells with HLA loss of heterozygosity in vitro and in vivo. Sci Transl Med.

[96] Wang X, Rivière I (2016). Clinical manufacturing of CAR T cells: foundation of a promising therapy. Mol Ther Oncolytics.

[97] Ellis J (2005). Silencing and variegation of gammaretrovirus and lentivirus vectors. Hum Gene Ther.

[98] Zhao Y, Moon E, Carpenito C, Paulos CM, Liu X, Brennan AL (2010). Multiple injections of electroporated autologous T cells expressing a chimeric antigen receptor mediate regression of human disseminated tumor. Cancer Res.

[99] Ramanayake S, Bilmon I, Bishop D, Dubosq M, Blyth E, Clancy L (2015). Low-cost generation of good manufacturing practice–grade CD19-specific chimeric antigen receptor–expressing T cells using piggyBac gene transfer and patient-derived materials. Cytotherapy.

[100] He J, Zhang Z, Lv S, Liu X, Cui L, Jiang D (2018). Engineered CAR T cells targeting mesothelin by piggyBac transposon system for the treatment of pancreatic cancer. Cell Immunol.

[101] Parkhurst MR, Yang JC, Langan RC, Dudley ME, Nathan DN, Feldman SA (2011). T cells targeting carcinoembryonic antigen can mediate regression of metastatic colorectal cancer but induce severe transient colitis. Mol Ther.

[102] Cherkassky L, Hou Z, Amador-Molina A, Adusumilli PS (2022). Regional CAR T cell therapy: an ignition key for systemic immunity in solid tumors. Cancer Cell.

[103] Adusumilli PS, Cherkassky L, Villena-Vargas J, Colovos C, Servais E, Plotkin J (2014). Regional delivery of mesothelin-targeted CAR T cell therapy generates potent and long-lasting CD4-dependent tumor immunity. Sci Transl Med.

[104] Singha NC, Nekoroski T, Zhao C, Symons R, Jiang P, Frost GI (2015). Tumor-associated hyaluronan limits efficacy of monoclonal antibody therapy. Mol Cancer Ther.

[105] Chanmee T, Ontong P, Itano N (2016). Hyaluronan: a modulator of the tumor microenvironment. Cancer Lett.

[106] Liu M, Tolg C, Turley E (2019). Dissecting the dual nature of hyaluronan in the tumor microenvironment. Front Immunol.

[107] Gmachl M, Sagan S, Ketter S, Kreil G (1993). The human sperm protein PH-20 has hyaluronidase activity. Febs Lett.

[108] Zhao R, Cui Y, Zheng Y, Li S, Lv J, Wu Q (2021). Human hyaluronidase PH20 potentiates the antitumor activities of mesothelin-specific CAR-T cells against gastric cancer. Front Immunol.

[109] Zhu Y, Zuo D, Wang K, Lan S, He H, Chen L (2023). Mesothelin-targeted CAR-T therapy combined with irinotecan for the treatment of solid cancer. J Cancer Res Clin.

[110] Moon EK, Wang L, Dolfi DV, Wilson CB, Ranganathan R, Sun J (2014). Multifactorial T-cell hypofunction that is reversible can limit the efficacy of chimeric antigen receptor-transduced human T cells in solid tumors. Clin Cancer Res.

[111] Hu W, Zi Z, Jin Y, Li G, Shao K, Cai Q (2019). CRISPR/Cas9-mediated PD-1 disruption enhances human mesothelin-targeted CAR T cell effector functions. Cancer Immunol Immunother.

[112] Jafarzadeh L, Masoumi E, Mirzaei HR, Alishah K, Fallah-Mehrjardi K, Khakpoor-Koosheh M (2021). Targeted knockdown of Tim3 by short hairpin RNAs improves the function of anti-mesothelin CAR T cells. Mol Immunol.

[113] Newick K, O'Brien S, Sun J, Kapoor V, Maceyko S, Lo A (2016). Augmentation of CAR T-cell trafficking and antitumor efficacy by blocking protein kinase A localization. Cancer Immunol Res.

[114] Masoumi E, Jafarzadeh L, Mirzaei HR, Alishah K, Fallah-Mehrjardi K, Rostamian H (2020). Genetic and pharmacological targeting of A2a receptor improves function of anti-mesothelin CAR T cells. J Exp Clin Canc Res.

[115] Li K, Xu J, Wang J, Lu C, Dai Y, Dai Q (2023). Dominant-negative transforming growth factor-β receptor-armoured mesothelin-targeted chimeric antigen receptor T cells slow tumour growth in a mouse model of ovarian cancer. Cancer Immunol Immunother.

[116] Adachi K, Kano Y, Nagai T, Okuyama N, Sakoda Y, Tamada K (2018). IL-7 and CCL19 expression in CAR-T cells improves immune cell infiltration and CAR-T cell survival in the tumor. Nat Biotechnol.

[117] Goto S, Sakoda Y, Adachi K, Sekido Y, Yano S, Eto M (2021). Enhanced anti-tumor efficacy of IL-7/CCL19-producing human CAR-T cells in orthotopic and patient-derived xenograft tumor models. Cancer Immunol Immunother.

[118] Liang Z, Dong J, Yang N, Li S, Yang Z, Huang R (2021). Tandem CAR-T cells targeting FOLR1 and MSLN enhance the antitumor effects in ovarian cancer. Int J Biol Sci.

[119] Moon EK, Carpenito C, Sun J, Wang LS, Kapoor V, Predina J (2011). Expression of a functional CCR2 receptor enhances tumor localization and tumor eradication by retargeted human T cells expressing a mesothelin-specific chimeric antibody receptor. Clin Cancer Res.

[120] Wang Y, Wang J, Yang X, Yang J, Lu P, Zhao L (2021). Chemokine receptor CCR2b enhanced anti-tumor function of chimeric antigen receptor T cells targeting mesothelin in a non-small-cell lung carcinoma model. Front Immunol.

[121] Mcgray AJR, Hallett R, Bernard D, Swift SL, Zhu Z, Teoderascu F (2014). Immunotherapy-induced CD8^+^ T cells instigate immune suppression in the tumor. Mol Ther.

[122] Cherkassky L, Morello A, Villena-Vargas J, Feng Y, Dimitrov DS, Jones DR (2016). Human CAR T cells with cell-intrinsic PD-1 checkpoint blockade resist tumor-mediated inhibition. J Clin Investig.

[123] Zolov SN, Rietberg SP, Bonifant CL (2018). Programmed cell death protein 1 activation preferentially inhibits CD28.CAR-T cells. Cytotherapy.

[124] Merighi S, Mirandola P, Varani K, Gessi S, Leung E, Baraldi PG (2003). A glance at adenosine receptors: novel target for antitumor therapy. Pharmacol Ther.

[125] Gessi S, Merighi S, Sacchetto V, Simioni C, Borea PA (1808). Adenosine receptors and cancer. Biochim Biophys Acta.

[126] Renner K, Singer K, Koehl GE, Geissler EK, Peter K, Siska PJ (2017). Metabolic hallmarks of tumor and immune cells in the tumor microenvironment. Front Immunol.

[127] Wang D, Dubois RN (2010). Eicosanoids and cancer. Nat Rev Cancer.

[128] Zelenay S, van der Veen AG, Böttcher JP, Snelgrove KJ, Rogers N, Acton SE (2015). Cyclooxygenase-dependent tumor growth through evasion of immunity. Cell.

[129] Huang S, Apasov S, Koshiba M, Sitkovsky M (1997). Role of A2a extracellular adenosine receptor-mediated signaling in adenosine-mediated inhibition of T-cell activation and expansion. Blood.

[130] Brudvik KW, Taskén K (2012). Modulation of T cell immune functions by the prostaglandin E2-cAMP pathway in chronic inflammatory states. Brit J Pharmacol.

[131] Jarnaess E, Taskén K (2007). Spatiotemporal control of cAMP signalling processes by anchored signalling complexes. Biochem Soc T.

[132] Ruppelt A, Mosenden R, Gronholm M, Aandahl EM, Tobin D, Carlson CR (2007). Inhibition of T cell activation by cyclic adenosine 5′-monophosphate requires lipid raft targeting of protein kinase A type I by the A-kinase anchoring protein Ezrin. J Immunol.

[133] Sitkovsky MV, Kjaergaard J, Lukashev D, Ohta A (2008). Hypoxia-adenosinergic immunosuppression: tumor protection by T regulatory cells and cancerous tissue hypoxia. Clin Cancer Res.

[134] Liu G, Zhang Q, Liu G, Li D, Zhang L, Gu Z (2021). Disruption of adenosine 2A receptor improves the anti-tumor function of anti-mesothelin CAR T cells both in vitro and in vivo. Exp Cell Res.

[135] Zhao H, Wei J, Sun J (2020). Roles of TGF-β signaling pathway in tumor microenvirionment and cancer therapy. Int Immunopharmacol.

[136] Luther SA, Bidgol A, Hargreaves DC, Schmidt A, Xu Y, Paniyadi J (2002). Differing activities of homeostatic chemokines CCL19, CCL21, and CXCL12 in lymphocyte and dendritic cell recruitment and lymphoid neogenesis. J Immunol.

[137] Pang N, Shi J, Qin L, Chen A, Tang Y, Yang H (2021). IL-7 and CCL19-secreting CAR-T cell therapy for tumors with positive glypican-3 or mesothelin. J Hematol Oncol.

[138] Tugues S, Burkhard SH, Ohs I, Vrohlings M, Nussbaum K, Vom BJ (2015). New insights into IL-12-mediated tumor suppression. Cell Death Differ.

[139] Kohli K, Pillarisetty VG, Kim TS (2022). Key chemokines direct migration of immune cells in solid tumors. Cancer Gene Ther.

[140] Stasi AD, Angelis BD, Rooney CM, Zhang L, Mahendravada A, Foster AE (2009). T lymphocytes coexpressing CCR4 and a chimeric antigen receptor targeting CD30 have improved homing and antitumor activity in a Hodgkin tumor model. Blood.

[141] Craddock JA, Lu A, Bear A, Pule M, Brenner MK, Rooney CM (2010). Enhanced tumor trafficking of GD2 chimeric antigen receptor T cells by expression of the chemokine receptor CCR2b. J Immunother.

[142] Vonderheide RH (2020). CD40 agonist antibodies in cancer immunotherapy. Annu Rev Med.

[143] Beatty GL, Torigian DA, Chiorean EG, Saboury B, Brothers A, Alavi A (2013). A phase I study of an agonist CD40 monoclonal antibody (CP-870,893) in combination with gemcitabine in patients with advanced pancreatic ductal adenocarcinoma. Clin Cancer Res.

[144] Nowak AK, Cook AM, Mcdonnell AM, Millward MJ, Creaney J, Francis RJ (2015). A phase 1b clinical trial of the CD40-activating antibody CP-870,893 in combination with cisplatin and pemetrexed in malignant pleural mesothelioma. Ann Oncol.

[145] Zhang Y, Wang P, Wang T, Fang Y, Ding Y, Qian Q (2021). Chimeric antigen receptor T cells engineered to secrete CD40 agonist antibodies enhance antitumor efficacy. J Transl Med.

[146] Chong EA, Alanio C, Svoboda J, Nasta SD, Landsburg DJ, Lacey SF (2022). Pembrolizumab for B-cell lymphomas relapsing after or refractory to CD19-directed CAR T-cell therapy. Blood.

[147] Fang J, Sun Y, Guo X, Xie B, Wang H, Mao W (2020). Safety and efficacy of chimeric antigen receptor T cells modified to target mesothelin and express PD-1 antibodies in patients with relapsed/refractory solid cancers in a phase I trial. J Clin Oncol.

[148] Adusumilli PS, Zauderer MG, Rivière I, Solomon SB, Rusch VW, O'Cearbhaill RE (2021). A phase I trial of regional mesothelin-targeted CAR T-cell therapy in patients with malignant pleural disease, in combination with the anti-PD-1 agent pembrolizumab. Cancer Discov.

[149] Wang Z, Li N, Feng K, Chen M, Zhang Y, Liu Y (2021). Phase I study of CAR-T cells with PD-1 and TCR disruption in mesothelin-positive solid tumors. Cell Mol Immunol.

[150] Zhao Y, Liu Z, Li L, Wu J, Zhang H, Zhang H (2021). Oncolytic adenovirus: prospects for cancer immunotherapy. Front Microbiol.

[151] Watanabe K, Luo Y, Da T, Guedan S, Ruella M, Scholler J (2018). Pancreatic cancer therapy with combined mesothelin-redirected chimeric antigen receptor T cells and cytokine-armed oncolytic adenoviruses. JCI Insight.

[152] Li Y, Xiao F, Zhang A, Zhang D, Nie W, Xu T (2020). Oncolytic adenovirus targeting TGF-β enhances anti-tumor responses of mesothelin-targeted chimeric antigen receptor T cell therapy against breast cancer. Cell Immunol.

[153] Qualls D, Salles G (2021). Optimizing CAR T cell therapy in lymphoma. Hematol Oncol.

[154] Castelletti L, Yeo D, van Zandwijk N, Rasko JEJ (2021). Anti-mesothelin CAR T cell therapy for malignant mesothelioma. Biomark Res.

[155] Pfirschke C, Engblom C, Rickelt S, Cortez-Retamozo V, Garris C, Pucci F (2016). Immunogenic chemotherapy sensitizes tumors to checkpoint blockade therapy. Immunity.

[156] Srivastava S, Furlan SN, Jaeger-Ruckstuhl CA, Sarvothama M, Berger C, Smythe KS (2021). Immunogenic chemotherapy enhances recruitment of CAR-T cells to lung tumors and improves antitumor efficacy when combined with checkpoint blockade. Cancer Cell.

[157] Bailly C (2019). Irinotecan: 25 years of cancer treatment. Pharmacol Res.

[158] Haas AR, Tanyi JL, O’Hara MH, Gladney WL, Lacey SF, Torigian DA (2019). Phase I study of lentiviral-transduced chimeric antigen receptor-modified T cells recognizing mesothelin in advanced solid cancers. Mol Ther.

[159] Beatty GL, Haas AR, Maus MV, Torigian DA, Soulen MC, Plesa G (2014). Mesothelin-specific chimeric antigen receptor mRNA-engineered T cells induce antitumor activity in solid malignancies. Cancer Immunol Res.

[160] Beatty GL, O’Hara MH, Lacey SF, Torigian DA, Nazimuddin F, Chen F (2018). Activity of mesothelin-specific chimeric antigen receptor T cells against pancreatic carcinoma metastases in a phase 1 trial. Gastroenterology.

[161] Li H, Huang H, Zhang T, Feng H, Wang S, Zhang Y (2022). Apatinib: a novel antiangiogenic drug in monotherapy or combination immunotherapy for digestive system malignancies. Front Immunol.

[162] Fang J, Ding N, Guo X, Sun Y, Zhang Z, Xie B (2021). αPD-1-mesoCAR-T cells partially inhibit the growth of advanced/refractory ovarian cancer in a patient along with daily apatinib. J Immunother Cancer.

